# Invasive alien species of policy concerns show widespread patterns of invasion and potential pressure across European ecosystems

**DOI:** 10.1038/s41598-023-32993-8

**Published:** 2023-05-19

**Authors:** Chiara Polce, Ana Cristina Cardoso, Ivan Deriu, Eugenio Gervasini, Konstantinos Tsiamis, Olga Vigiak, Grazia Zulian, Joachim Maes

**Affiliations:** 1 European Commission, Joint Research Centre, Ispra, Italy; 2 ARHS Developments S.A., Luxembourg, Luxembourg; 3 European Commission, Brussels, Belgium; 4 Seidor Italy SRL, Milan, Italy

**Keywords:** Ecosystem services, Invasive species, Biogeography, Ecology

## Abstract

Animals, plants, and other organisms unintentionally or deliberately brought into a natural environment where they are not normally found, and where they cause harmful effects on that environment, are known also as invasive alien species (IAS). They represent a major threat to native biodiversity and ecosystem functioning, and can affect negatively human health and the economy. We assessed the presence and potential pressure by IAS on terrestrial and freshwater ecosystems across 27 European countries, for 66 IAS of policy concern. We computed a spatial indicator that accounts for the number of IAS present in an area and the extent of the ecosystems affected; for each ecosystem, we also looked at the pattern of invasions in the different biogeographical regions. We found disproportionally greater invasion in the Atlantic region, followed by Continental and Mediterranean regions, possibly related to historical patterns of first introductions. Urban and freshwater ecosystems were the most invaded (nearly 68% and ca. 52% of their extent respectively), followed by forest and woodland (nearly 44%). The average potential pressure of IAS was greater across cropland and forests, where we also found the lowest coefficient of variation. This assessment can be repeated over time to derive trends and monitor progress towards environmental policy objectives.

## Introduction

Alien species are living organisms (e.g. animals, plants, fungi, microorganisms) introduced unintentionally or consciously into a new environment outside their natural geographic range. Humans have deliberately moved species throughout history to meet their basic requirements or to enhance the quality of their existence: for instance, crop species and farmed animals, or ornamental plants and animal introduced as pets or as game, or species introduced for biological control of pests. Some of those species have afterwards established in the wild. Human activities are also responsible for unintentional introductions, for instance translocations of species because of transport or artificial corridors. Typical examples are organisms transported in ballast water, like the zebra mussel (*Dreissena polymorpha*)^1,2^, or non-indigenous vectors of diseases responsible, for instance, for outbreaks of malaria, yellow fever, typhus, and plague^3^.

In new environments, many alien species lack their natural enemies or other limiting factors, such as food, space or competition with other species, all conditions that can favor their establishment and rapid spread^4,5^. Thus, alien species have the potential to become invasive (IAS), displace and cause the loss of native species, alter habitats, change community structure and interfere with food-web relationships and ecosystem processes^6–9^. Furthermore, many alien species can carry diseases^10,11^ exacerbating the potential threat to local biodiversity, human health and the economy^12^.

New alien species are likely to be transported from their native regions to new areas, due to the increased movement of goods and people. Efforts should aim at identifying and controlling introduction pathways, eradicating invaders in their early stages, and stopping the spread of previously introduced species. As a result, it is necessary to take coordinated preventative and management measures to lessen the likelihood of new introductions and the damage they could cause. Article 8 (h) of the Convention of Biological Diversity^13^ states that “Each Contracting Party shall, as far as possible and as appropriate: […] Prevent the introduction of, control or eradicate those alien species which threaten ecosystems, habitats or species”. The persistence and magnitude of this pressure are addressed also by Aichi target 9^14^.

Recent global assessments^15,16^ concluded that Aichi target 9 was only partially achieved. In particular, while the prioritization of IAS was found satisfactory, progress towards other elements of the target, such as pathways prioritization and management, and species control and eradication, was found insufficient or with limited information. Therefore, IAS continue to increase globally and remain one of the primary threats to biodiversity.

In the European Union (EU), the EU Regulation 1143/2014^17^ “on the prevention and management of the introduction and spread of invasive alien species” (hereinafter referred to as the IAS Regulation) requires the European Commission to adopt and update as needed, a list of IAS of Union concern ('the Union list'). The Union list is based on risk assessments of IAS, which include, among other elements, documented evidence about the species’ ability to establish and spread. At the European Union level, coordinated actions are taken toward IAS on the Union list. Additionally, the IAS Regulation requires the implementation of eradication measures, unless a cost–benefit analysis demonstrates that the associated costs are disproportionate to the benefits, in which case containment measures must be adopted. To minimize the impacts of widely distributed IAS on biodiversity, ecosystem services, and if applicable, on human health or the economy, efficient management measures are also required.

The first Union list entered into force in 2016 with 37 species^18^, while the 2017 and 2019 updates added respectively 12 and 17 species^19,20^. Some of the species on the Union list figure amongst the world’s worst IAS^21^ such as the Water hyacinth (*Eichhornia crassipes*), the Chinese mitten crab (*Eriocheir sinensis*), the Red-eared, Yellow-bellied and Cumberland slider (different subspecies of *Trachemys scripta*), the Grey squirrel (*Sciurus carolinensis*) and the Small Asian mongoose (*Herpestes javanicus*). Out-competition and predation of native biodiversity, impairment of ecosystem function, and damage to agricultural systems and human infrastructure are all examples of their impacts.

For the first time to our knowledge, here we provide an assessment of the presence coupled with the potential pressures caused by IAS on terrestrial and freshwater ecosystems across the member states of the EU (27 members in 2022), for 66 IAS on the Union list following the 2019 update^20^. This update includes 36 plant and 30 animal species (Suppl. Tables S1 and S2). From the assessment are excluded the outermost regions of the EU, where specific lists of IAS of concern are adopted, following Art. 6 of the EU Regulation 1143/2014^17^.

The potential pressure was assessed through an indicator that accounts for the number of IAS present in an area and the extent of the ecosystem(s) affected. Our assessment is not yet an indicator of damage (negative impact), as the damage an IAS causes will also depend on the susceptibility of an ecosystem to one or more IAS and the bioclimatic conditions. This approach, however, allowed us to assess both the distribution and the magnitude of the potential pressure caused by IAS across terrestrial and freshwater ecosystems.

We followed the ecosystem typology adopted for the EU ecosystem assessment^22^, developed by Maes et al.^23^ to map and assess the ecosystems and their services across the European Union.

Given the broad bioclimatic variability characterizing the EU, for each ecosystem type we also looked at the pattern of invasions in the different biogeographical regions (hereinafter bioregions) (Suppl. Fig. S1). Specifically, we compared the relative extent of each bioregion with the relative extent invaded by IAS.

## Results

### Potential pressure across all ecosystems and biogeographical regions

**Figure 1:**
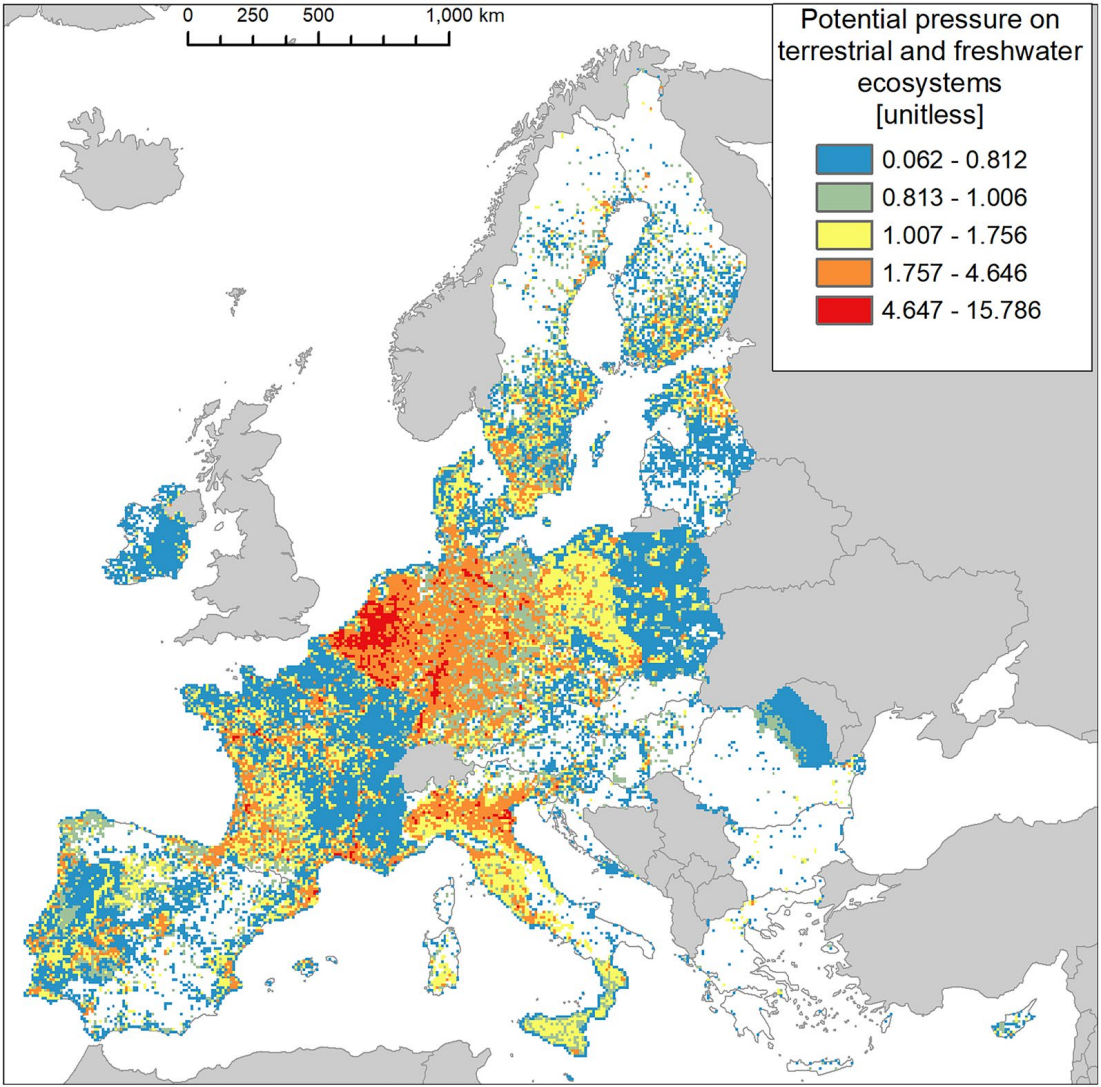
Cumulative potential pressure by invasive alien species (IAS) of Union concern across terrestrial and freshwater ecosystems for the 27 member states of the European Union (EU), with the exclusion of the outermost regions. Countries beyond the geographic scope of the study are in light grey. Values are grouped in geometric intervals. Potential pressure is computed solely for areas where IAS are reported. White mainland indicates areas where IAS are not reported.

Figure 1 shows the cumulative potential pressure across all ecosystems (terrestrial and freshwater). The values indicate the total potential pressure by IAS reported in each reference area (a 100-km^2^ square of the reference grid). Large areas of greater potential pressure are visible across Belgium and Netherlands, the western part of Germany, the northern part of Italy and the Mediterranean and western Atlantic coast of France. Large areas of relatively low potential pressure are visible across Ireland, France, Poland, and along the border between Romania and Moldova.

**Figure 2:**
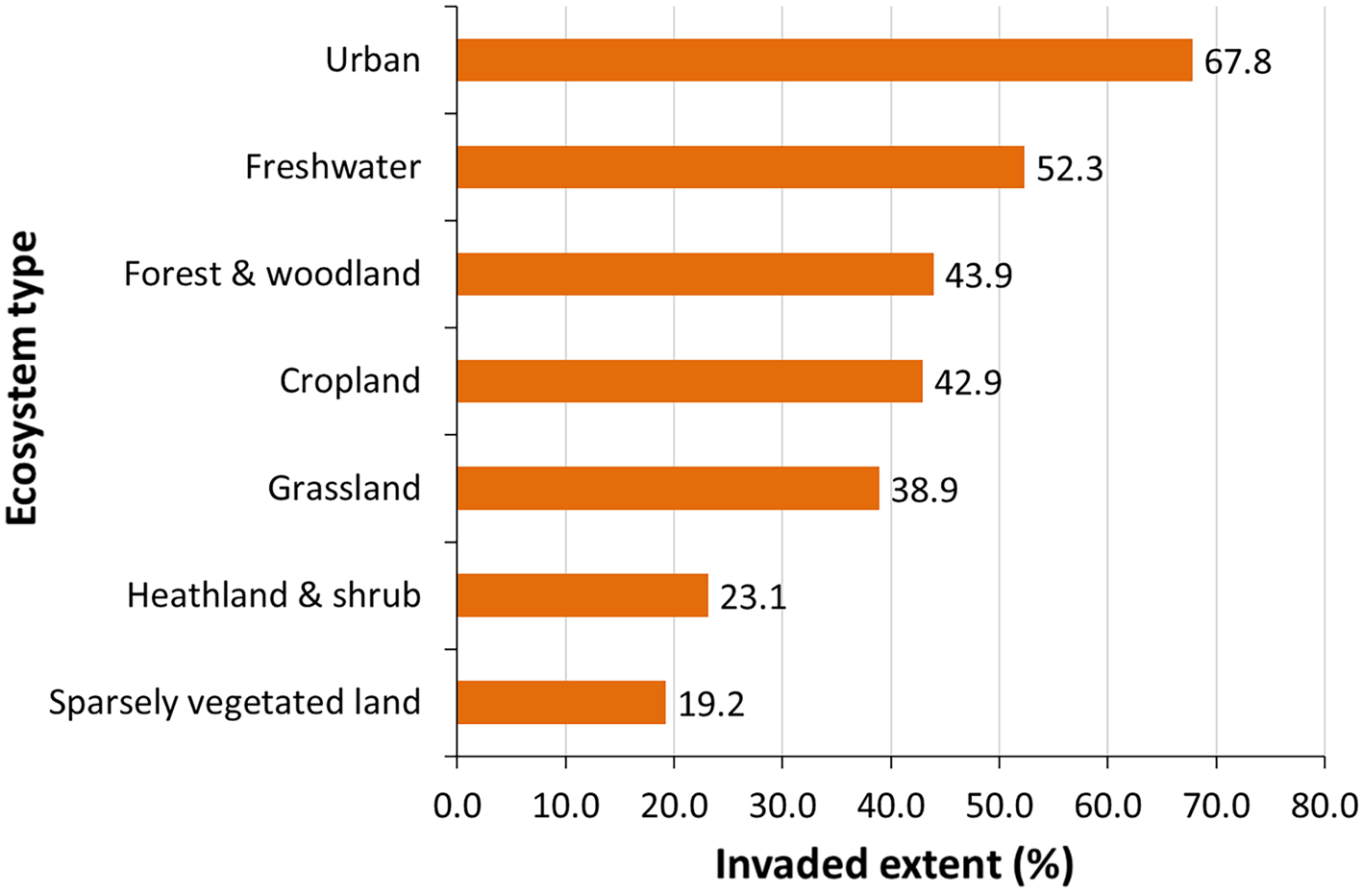
Invaded areas across ecosystem types, as a percent of their total extent.

Figure 2 shows invaded areas as the percent of each ecosystem’s total extent. Urban and freshwater ecosystems are invaded for more than 50% of their total area (67.8 and 52.3% respectively), whilst heathland and shrub, and sparsely vegetated land for less than 25% (23.1 and 19.2% respectively).

**Figure 3:**
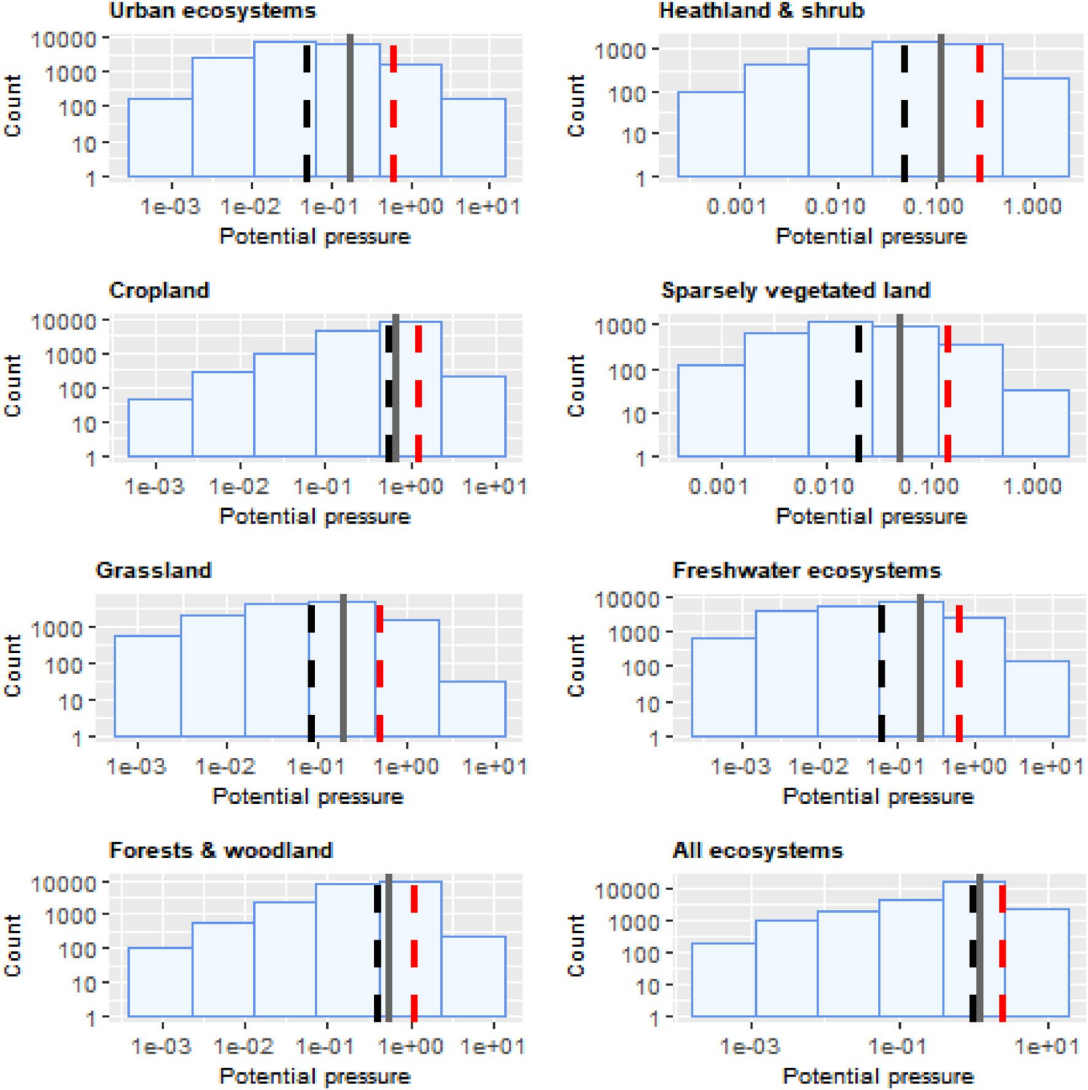
Frequency of potential pressure values across ecosystems and for all ecosystems, in areas invaded by IAS, on a log–log scale. To complement the patterns, arithmetic mean (grey solid lines), median (black dashed lines) and standard deviation (red dashed lines) are superimposed (see also Suppl. Table S3).

The histograms of potential pressure for each ecosystem and for all ecosystem types together are in Fig. 3. For all ecosystem types, the minimum potential pressure was 0.001, while the maximum ranged from 1.247 in sparsely vegetated land to 10.859 in freshwater ecosystems (Suppl. Table S3). Considering the risk that a species on the list is such that eradication is required when detected, possibly anything above 0.001 (more than five ha) is already worrying.

The highest average potential pressure was recorded in cropland, followed by forest (0.657 and 0.543 respectively, Suppl. Table S3). The magnitude of the standard deviation observed across all ecosystems assessed, however, suggests that local conditions might sensibly influence the average pattern of invasion (Fig. 3 and Suppl. Table S3). The highest coefficient of variation was found in urban ecosystems (260.8%) whilst the lowest one in cropland (82.8%) (Suppl. Table S3).

**Figure 4:**
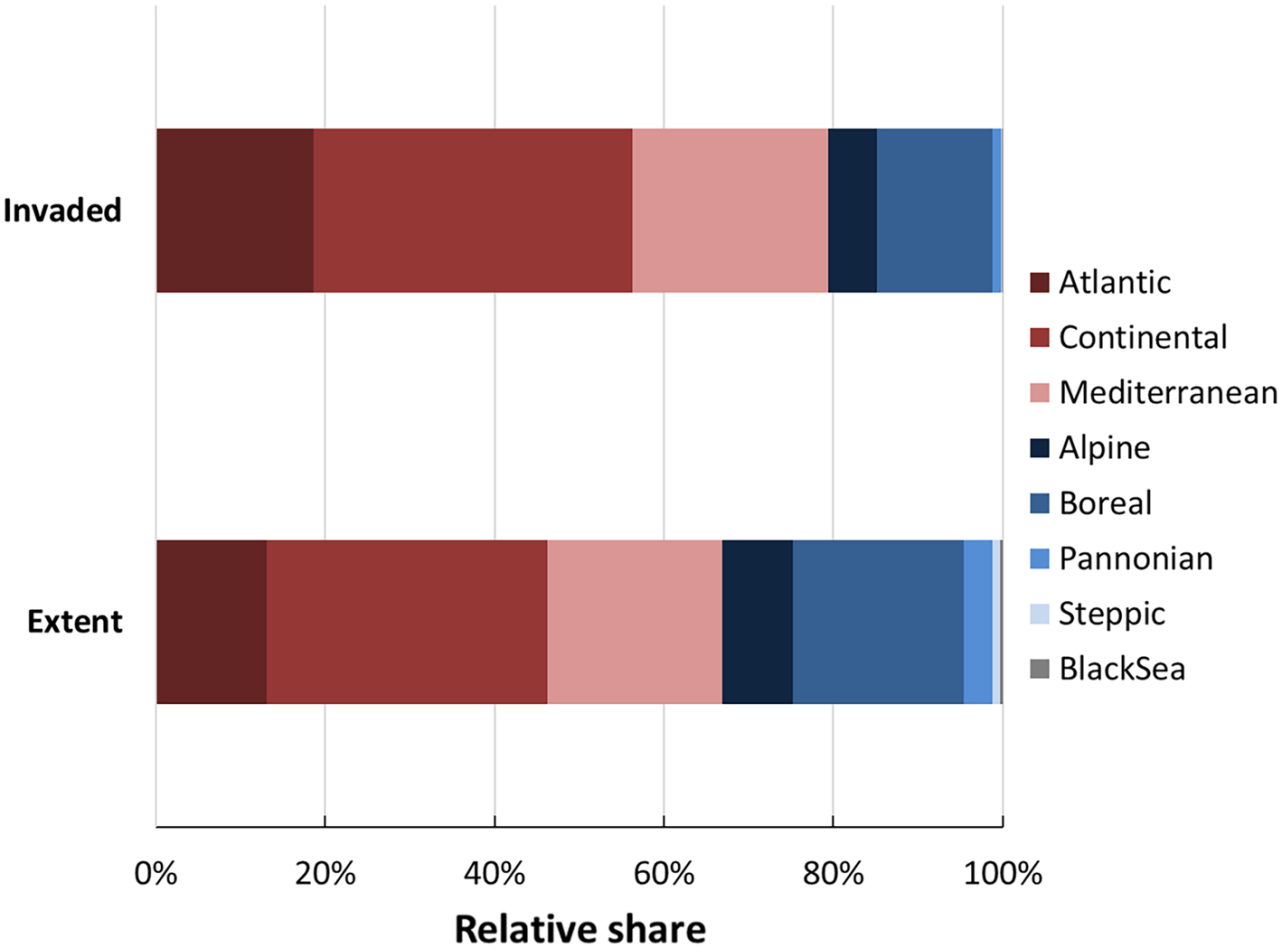
Biogeographical characterization of invaded areas compared to the relative extent of the biogeographical region. Regions are sorted in descending order: shades of red indicate regions that are invaded more than expected based on their relative extent (darker shades indicate greater-than-expected invasion); shades of blue indicate regions disproportionally less invaded, compared to their relative extent.

Figure 4 compares the biogeographical characterization of the invaded areas, to the relative extent of each biogeographical region. The figures shows that Atlantic, Continental and Mediterranean regions are invaded more than expected based on their relative extent.

### Potential pressure by ecosystem type

For each ecosystem type, we present a chart with its biogeographical composition and the related characterisation of invaded areas, to show their proportional differences; and a map of potential pressure by IAS showing the spatial pattern, across the 100-km^2^ reference grid. Potential pressure is computed solely for areas where IAS are reported and affecting the ecosystem. Outside these areas, the presence of the ecosystem on each relevant map is indicated in dark grey (‘Extent’), mapped on the original 100-m spatial resolution. Countries beyond the geographic scope of the study are in light grey. In the “Supplementary Information” we show, for each ecosystem type, the number of IAS across the invaded areas (i.e., the IAS richness), the relative extent of invaded areas (i.e., the extent of the ecosystem within the 100-km^2^ grid cell), and the relative invasion by each IAS (i.e., percent of invaded areas).

### Urban ecosystems

**Figure 5:**
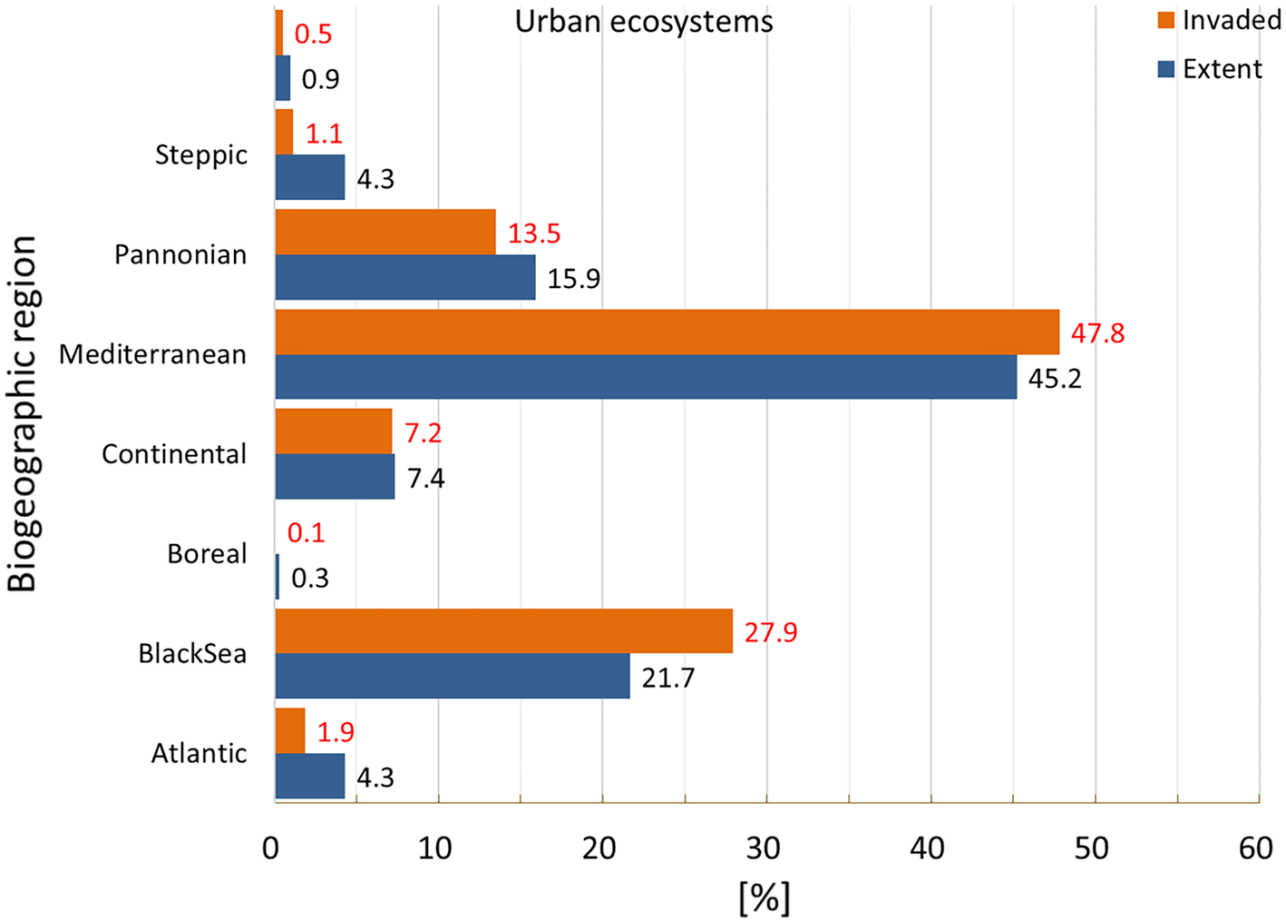
Biogeographic characterisation of urban ecosystems: extent versus invasion. Label on bars indicate the correspondent area as percent of the total extent (in blue) or as percent of the total invaded area (in orange).

Urban ecosystems include a variety of land features, which can act as habitats to many species, including aliens. Invasion was present in nearly 68% of the area occupied by urban ecosystems (Fig. 2), ranking this ecosystem type as the most invaded one. The biogeographical distribution of IAS showed a disproportionally greater prevalence in Atlantic and Continental regions (Fig. 5).

**Figure 6:**
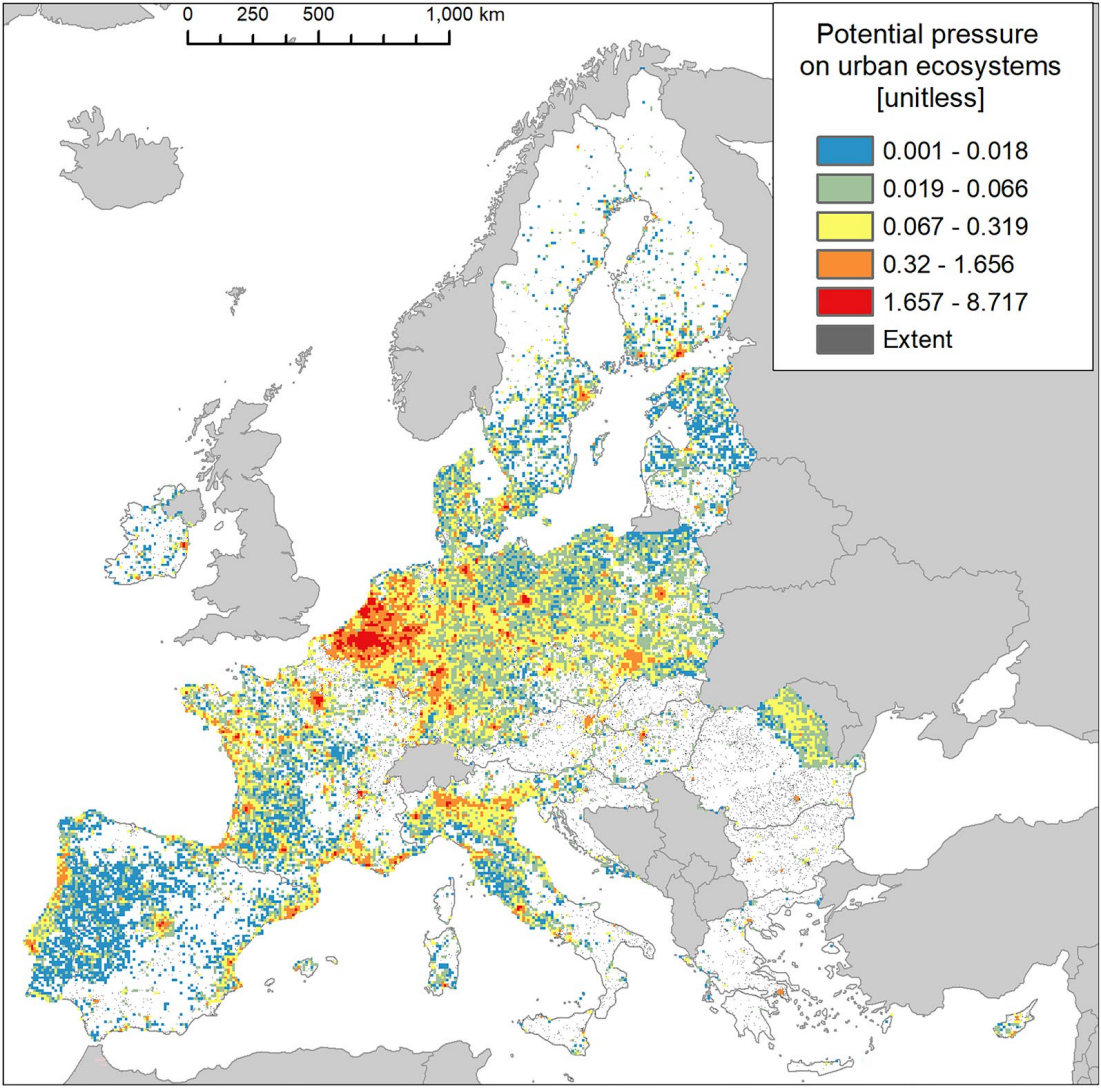
Potential pressure by IAS on urban ecosystems, mapped on the 100-km^2^ reference squares. Extent indicates areas of urban ecosystems without reporting of IAS (dark grey). Countries beyond the geographic scope of the study are in light grey. See main text for additional details.

Despite the maximum potential pressure was ca. 8.7 (Fig. 6) and the highest across terrestrial ecosystems, the shape of the histogram as well as the summary statistics indicate that most of the potential pressure was in the lowest range of values (Fig. 3 and Suppl. Table S3). Potential pressure in the highest end was widely recorded across Belgium, Netherlands and Germany, with isolated but noticeable spots across France, Italy, Portugal, Spain and the Scandinavian countries. Large areas of low values of potential pressure were found in Portugal, the western part of France, the central part of Italy, and across Latvia and Estonia (Fig. 6). The number of reported IAS, the relative extent of invaded areas, and the percent of invasion by each IAS are shown in the “Supplementary Information” (Fig. S2, Tables S5 and S6).

### Cropland ecosystems

**Figure 7:**
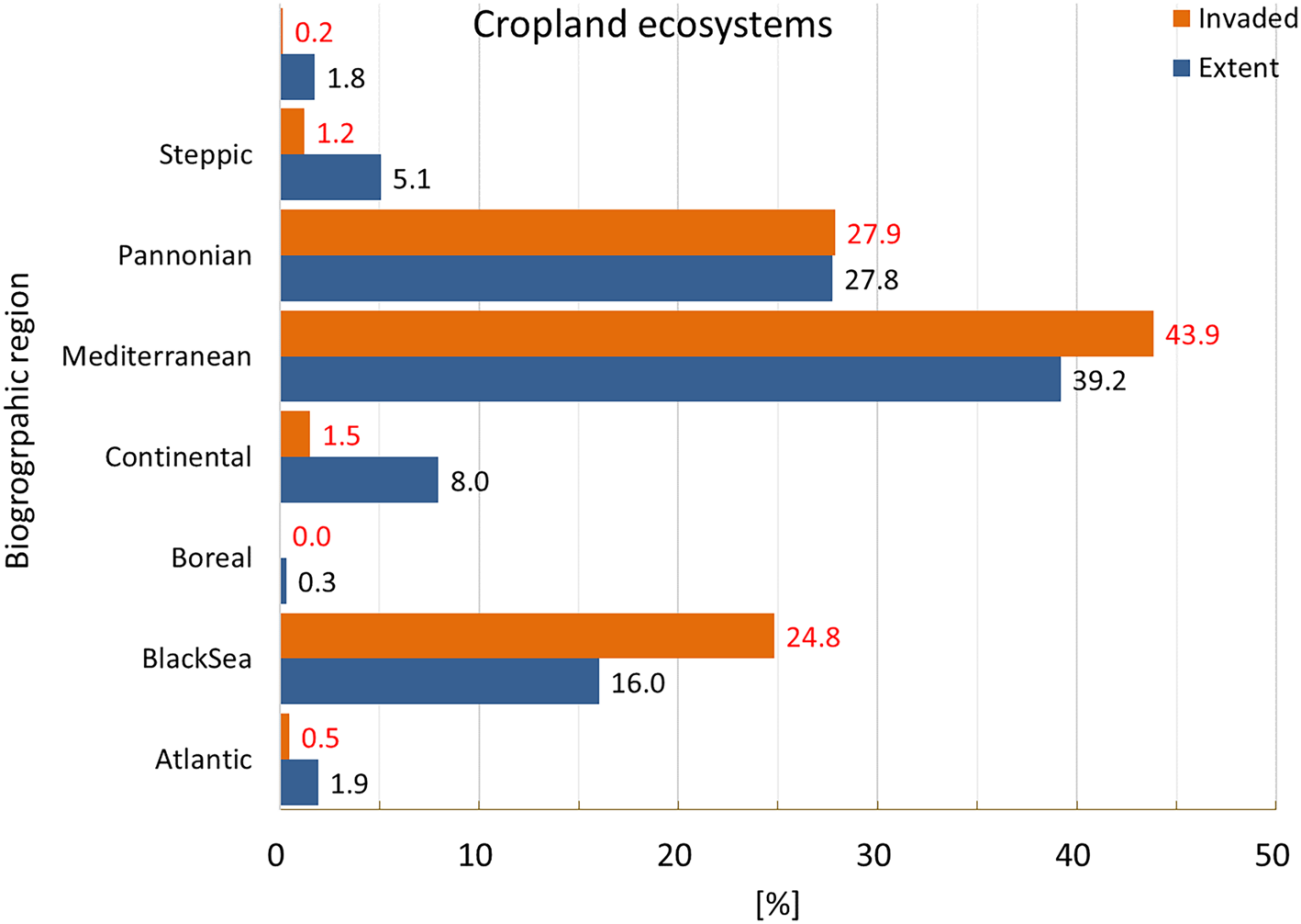
Biogeographic characterisation of cropland: extent versus invasion. Label on bars indicate the correspondent area as percent of the total extent (in blue) or as percent of the total invaded area (in orange).

Invasion was present in almost 43% of the area occupied by cropland (Fig. 2), with disproportionally greater prevalence in Atlantic, followed by Continental and Mediterranean regions (Fig. 7).

**Figure 8:**
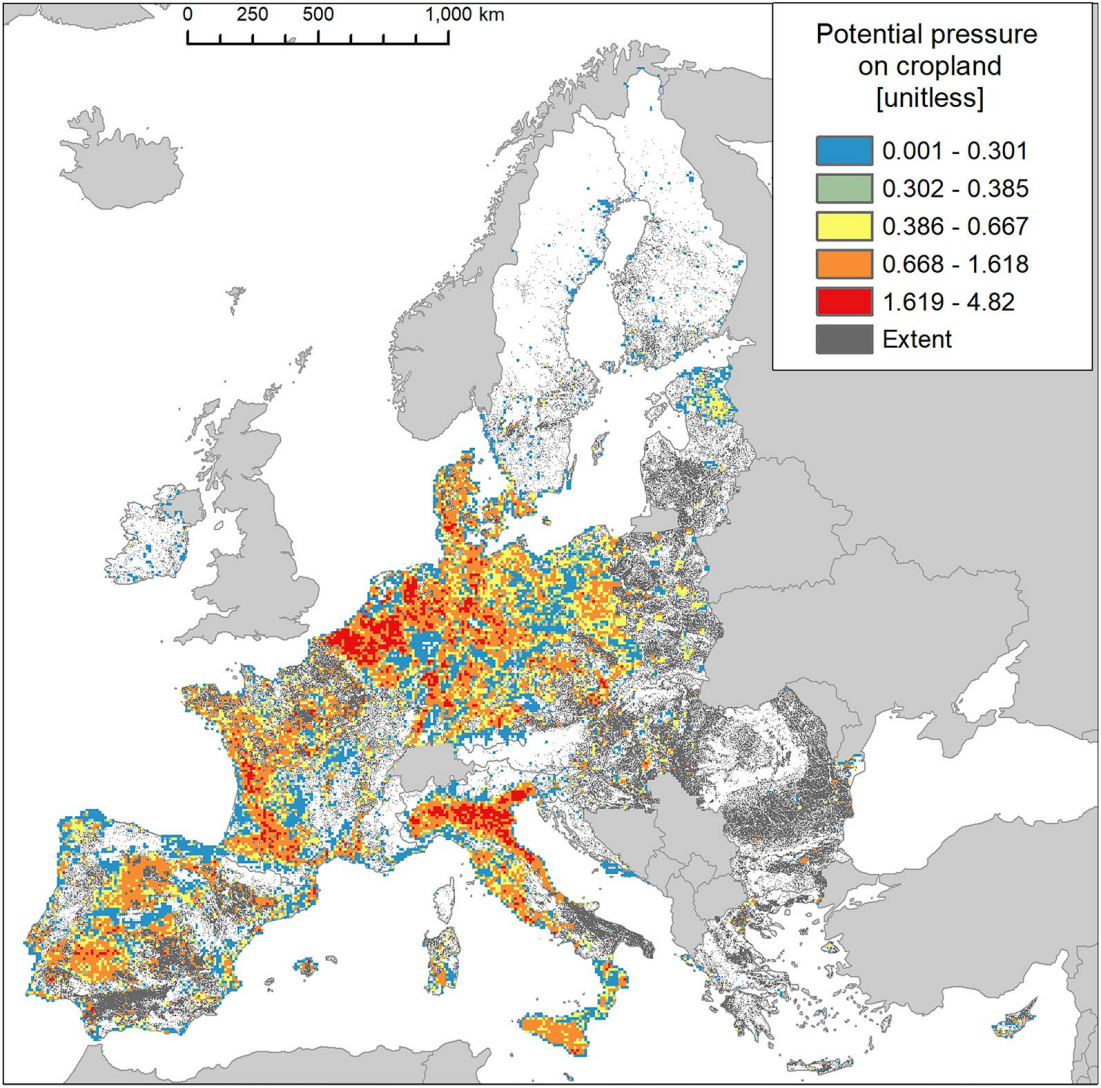
Potential pressure by IAS on cropland, mapped on the 100-km^2^ reference squares. Extent indicates areas of cropland without reporting of IAS (dark grey). Countries beyond the geographic scope of the study are in light grey. See main text for additional details.

Cropland was the ecosystem with the greatest mean potential pressure and the lowest coefficient of variation (mean ± SD: 0.657 ± 0.544; CV: 82.8%; Suppl. Table S3). While maximum potential pressure (ca. 4.8, Fig. 8) was not as high as in urban ecosystems, values were far less clumped to the lowest end than in urban ecosystems (Fig. 3 and Suppl. Table S3). Potential pressure in the highest end was noticeable across Italy (particularly in the North), Netherlands and Belgium. Large areas of high values potential pressure were widely recorded across the western part of Spain, France, Poland, Germany, Denmark and the island of Sicily in Italy. In most of these countries, however, widespread areas of low values potential pressure were also found (Fig. 8). The number of reported IAS, the relative extent of invaded areas, and the percent of invasion by each IAS are shown in the “Supplementary Information” (Fig. S3, Tables S7 and S8).

### Grassland ecosystems

**Figure 9:**
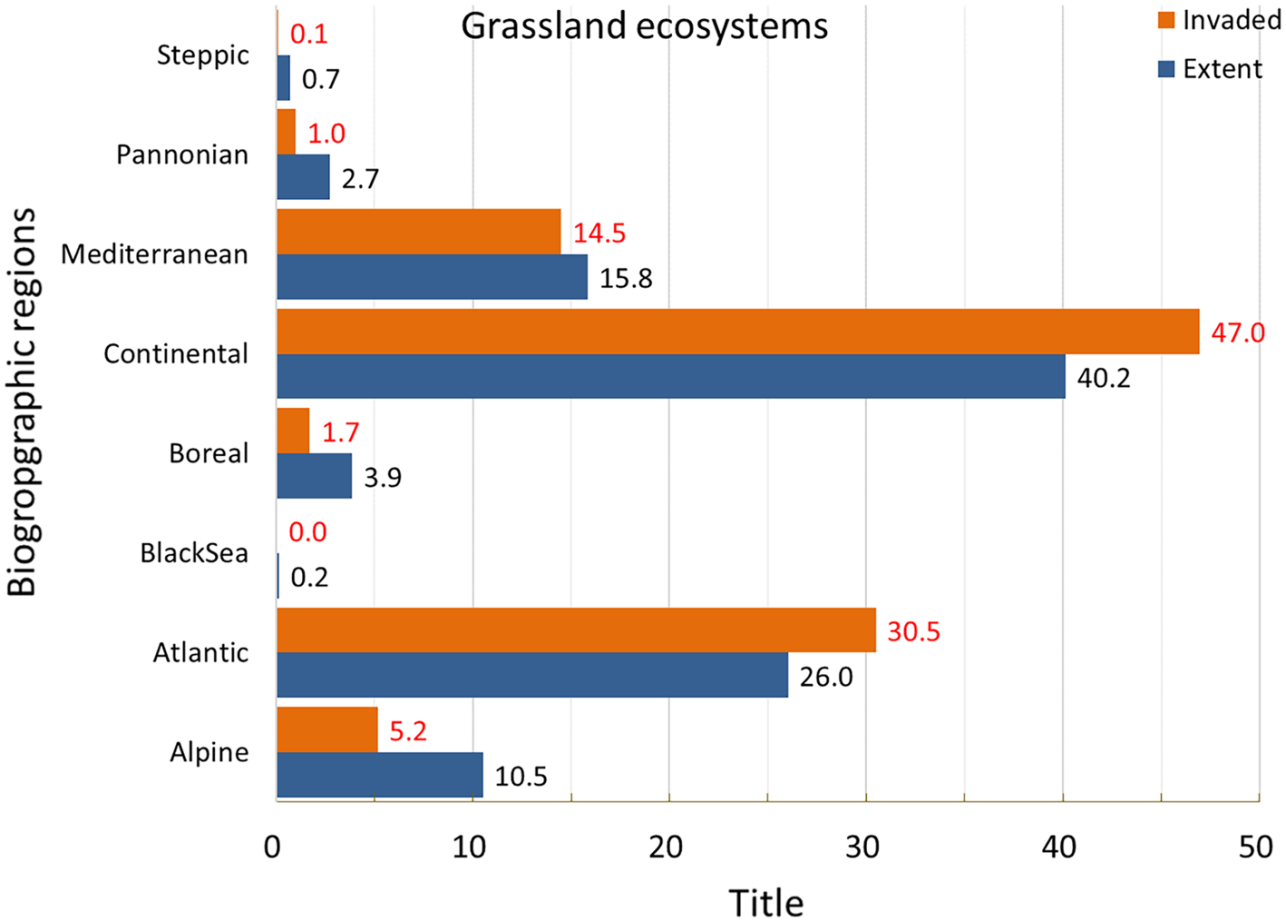
Biogeographic characterisation of grassland: extent versus invasion. Label on bars indicate the correspondent area as percent of total extent (in blue) or as percent of total invaded area (in orange).

Invasion was present in almost 39% of the area occupied by grassland (Fig. 2), with disproportionally greater prevalence in Continental and Atlantic regions (Fig. 9).

**Figure 10:**
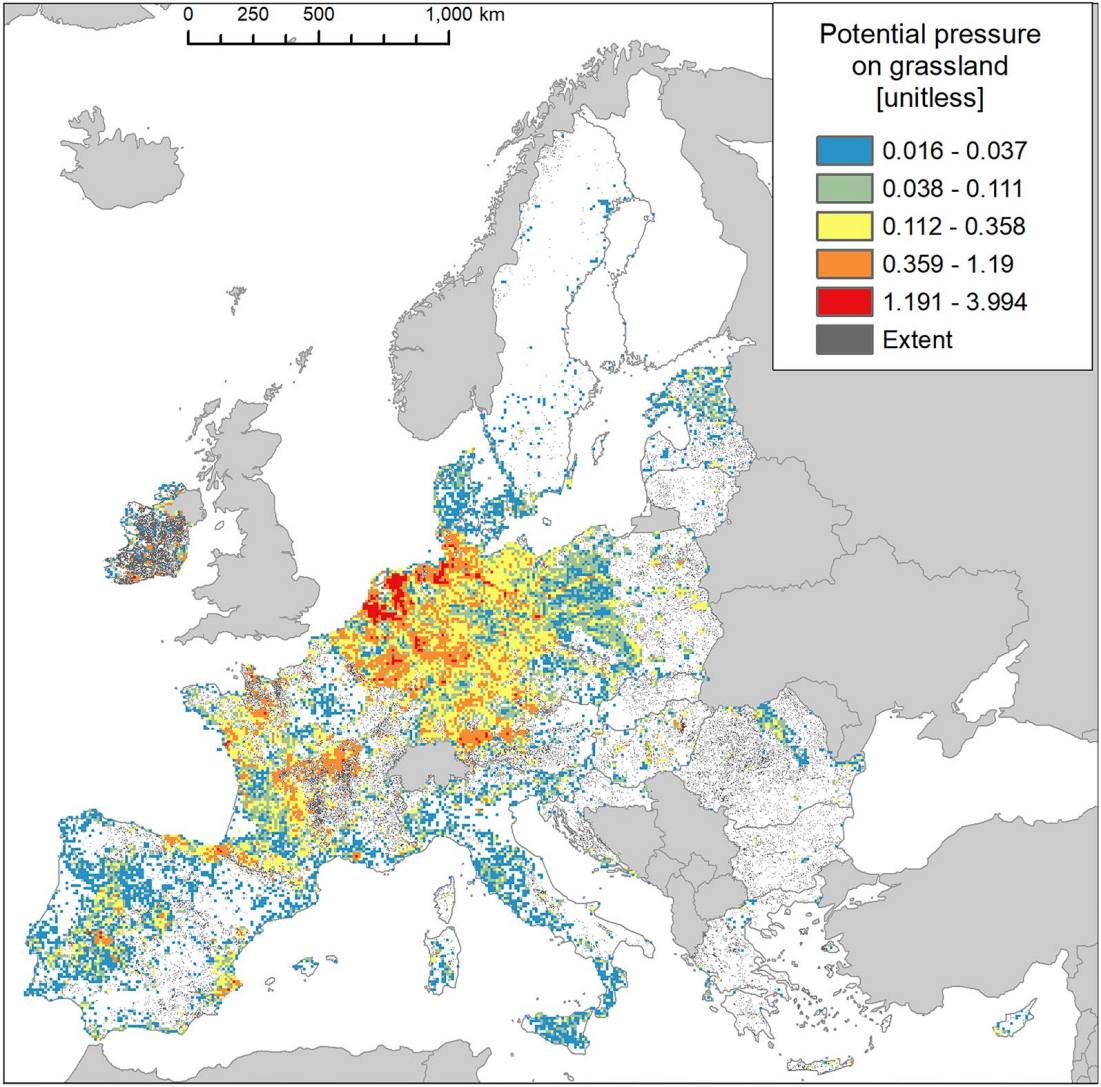
Potential pressure by IAS on grassland, mapped on the 100-km^2^ reference squares. Extent indicates areas of grassland without reporting of IAS (dark grey). Countries beyond the geographic scope of the study are in light grey. See main text for additional details.

Maximum potential pressure reached almost 4 (Fig. 10), with most values distributed around the lowest ranges (median: 0.084, Suppl. Table S3) and standard deviation ca. 1.5 time greater than the mean (Fig. 3 and Suppl. Table S3). Potential pressure in the highest range was recorded throughout Netherlands and the northern part of Germany. Large areas of low values potential pressure were obtained across Spain, Portugal, Italy; across Denmark, the western part of Poland, Czech Republic, and the Carpathian Mountains in Romania (Fig. 10). The number of reported IAS, the relative extent of invaded areas, and the percent of invasion by each IAS are shown in the “Supplementary Information” (Fig. S4, Tables S9 and S10).

### Forest and woodland ecosystems

**Figure 11:**
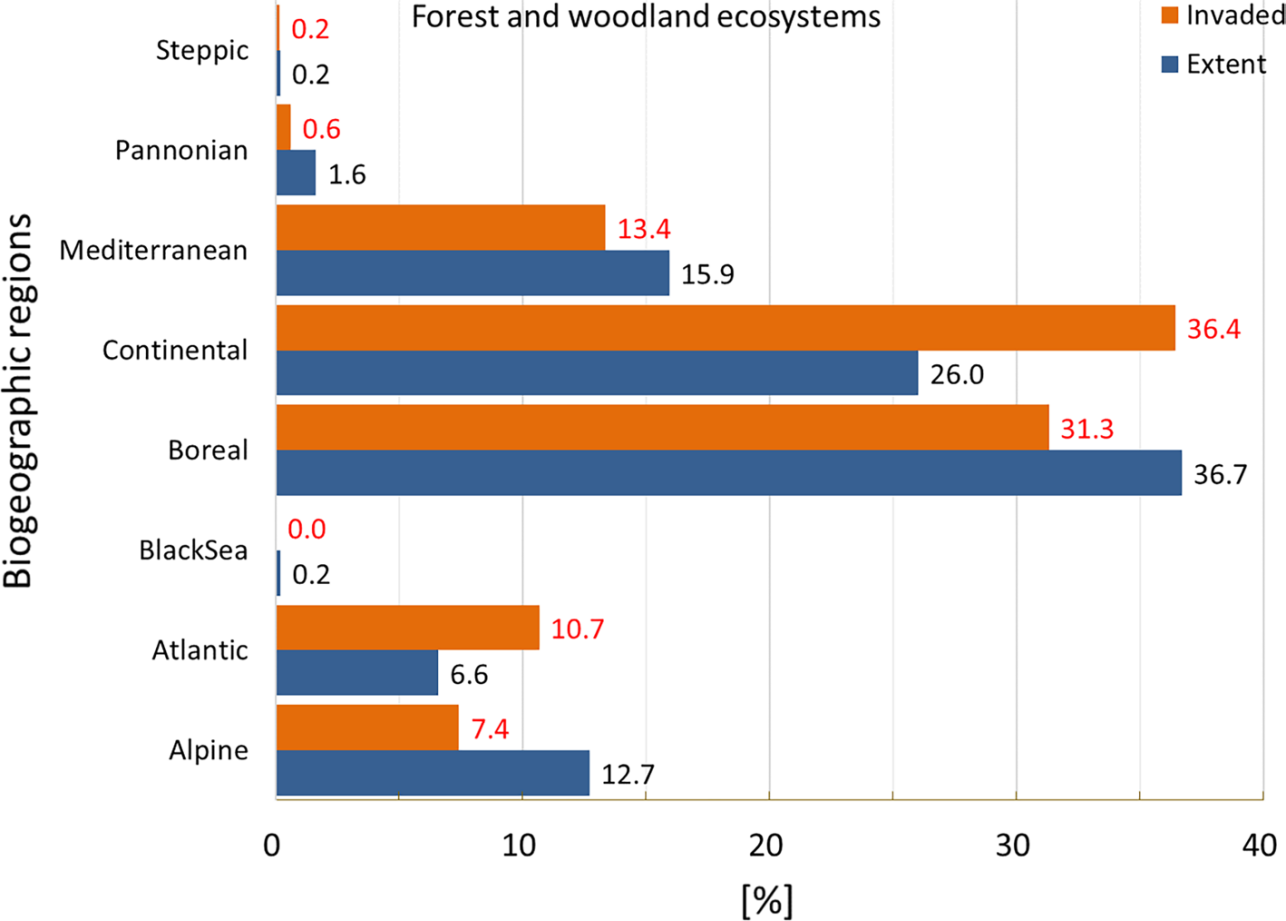
Biogeographic characterisation of forests: extent versus invasion. Label on bars indicate the correspondent area as percent of total extent (in blue) or as percent of the total invaded area (in orange).

Invasion was present in almost 44% of the area occupied by forest and woodland (hereafter forests), the second largest invaded terrestrial ecosystem after urban ecosystems (Fig. 2). Atlantic and Continental regions were disproportionally more invaded (Fig. 11).

**Figure 12:**
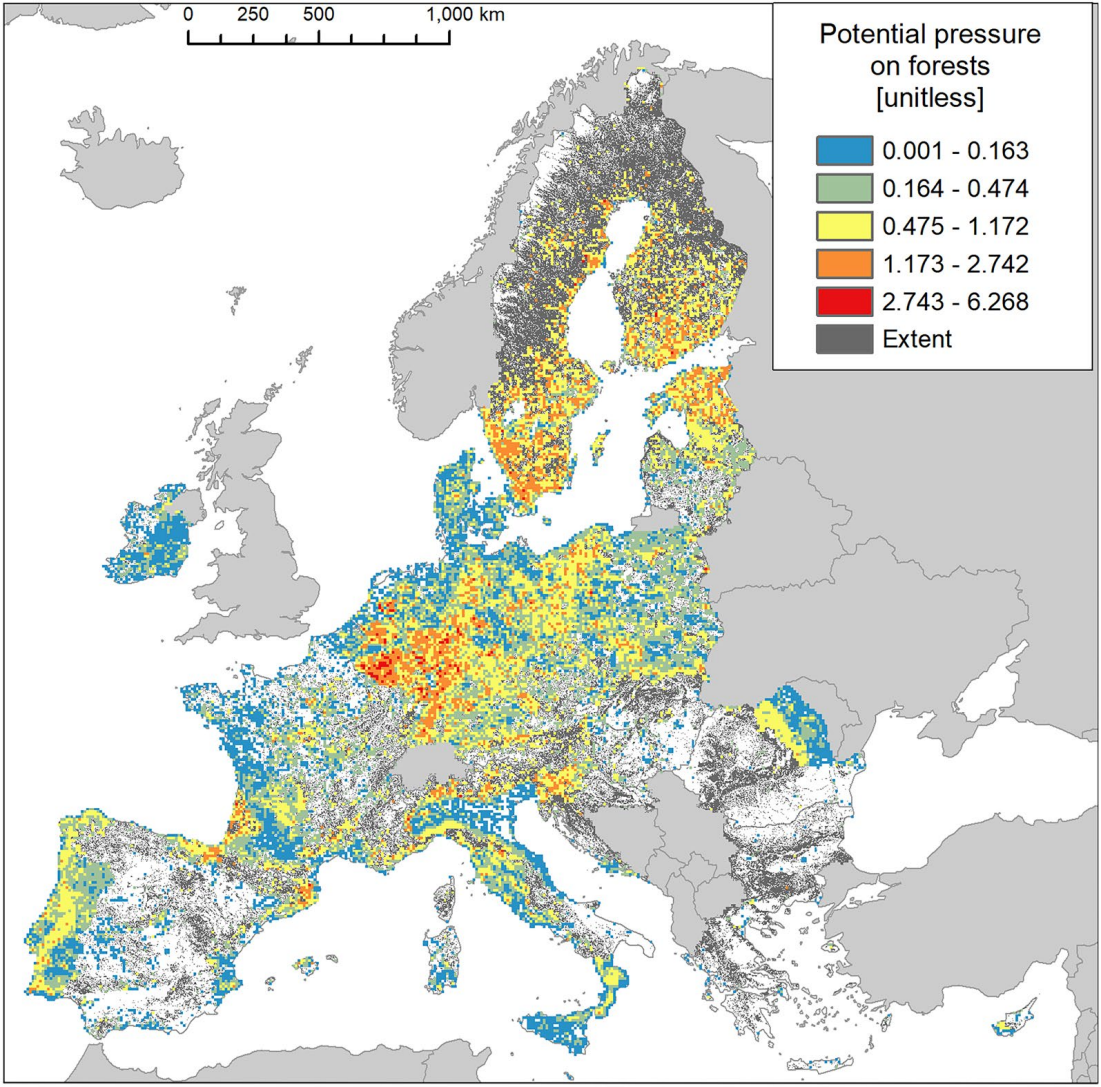
Potential pressure by IAS on forests, mapped on the 100-km^2^ reference squares. Extent indicates areas of forests without reporting of IAS (dark grey). Countries beyond the geographic scope of the study are in light grey. See main text for additional details.

In addition, forest was the ecosystem with the second largest mean potential pressure and the second lowest coefficient of variation after cropland (mean ± SD: 0.543 ± 0.529; CV: 97.4%; Suppl. Table S3). Maximum potential pressure was greater than 6 (Fig. 12), the second highest potential pressure recorded across terrestrial ecosystems, after urban ecosystems. As observed in cropland, the statistical distribution of potential pressure values showed a median near the mean (median: 0.407, Fig. 3 and Suppl. Table S3). Pressure in the highest ranges was recorded mainly across Belgium, Netherlands, and Germany, followed by Scandinavian countries and Estonia (Fig. 12). The number of reported IAS, the relative extent of invaded areas, and the percent of invasion by each IAS are shown in the “Supplementary Information” (Fig. S5, Tables S11 and S12).

### Heathland and shrub ecosystems

**Figure 13:**
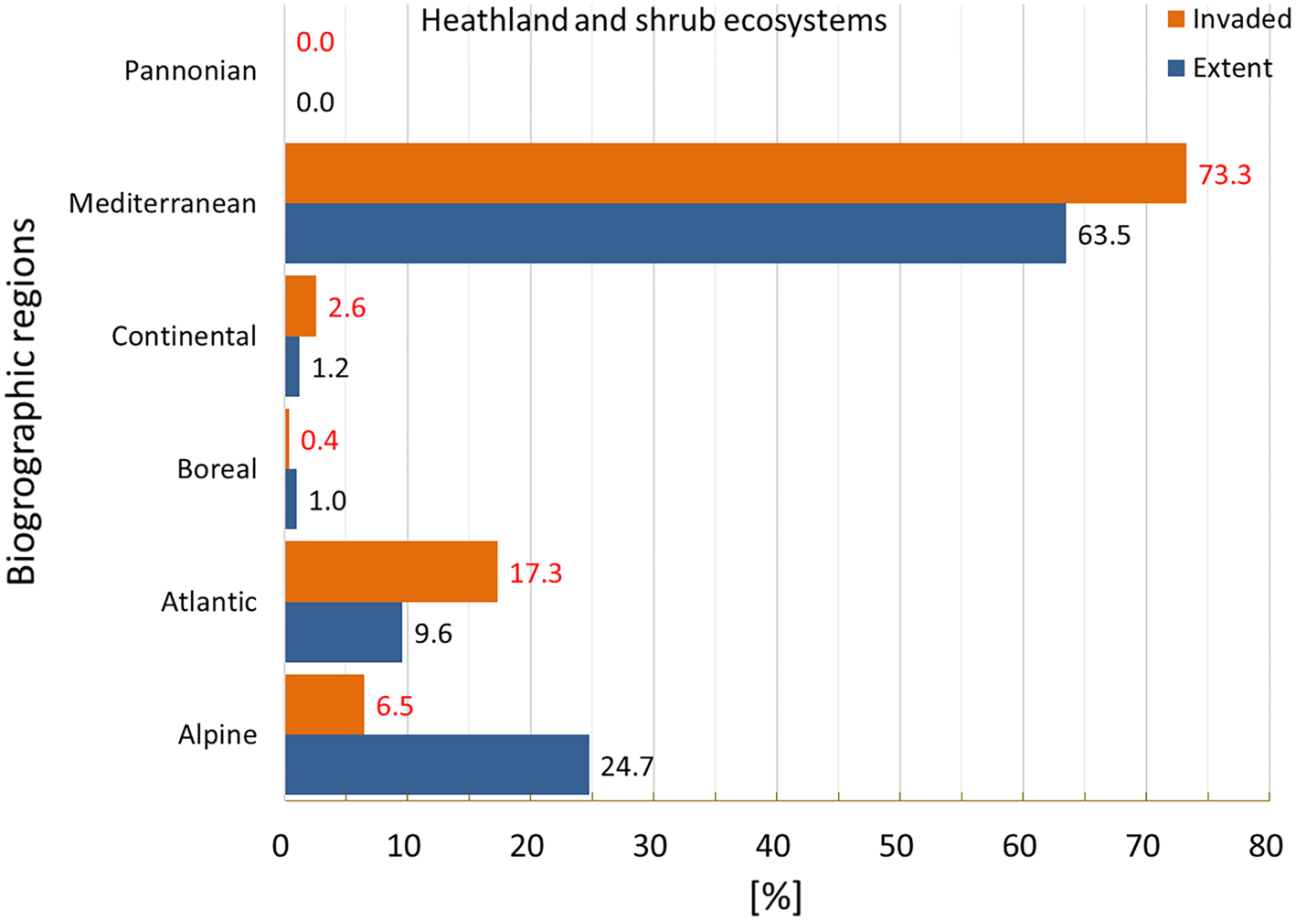
Biogeographic characterisation of heathland and shrub: extent versus invasion. Label on bars indicate the correspondent area as percent of the total extent (in blue) or as percent of the total invaded area (in orange).

Invasion was present just above 23% of heathland and shrub, placing this ecosystem among the ones least invaded (second after sparsely vegetated land, Fig. 2). Invasion affected disproportionally the Continental, Mediterranean and Atlantic regions (Fig. 13). The statistics showed large variation in potential pressure around the mean, and a median ca. 59% lower than the mean value (mean ± SD: 0.111 ± 0.165; CV: 149.4%; median: 0.046; Suppl. Table S3).

**Figure 14:**
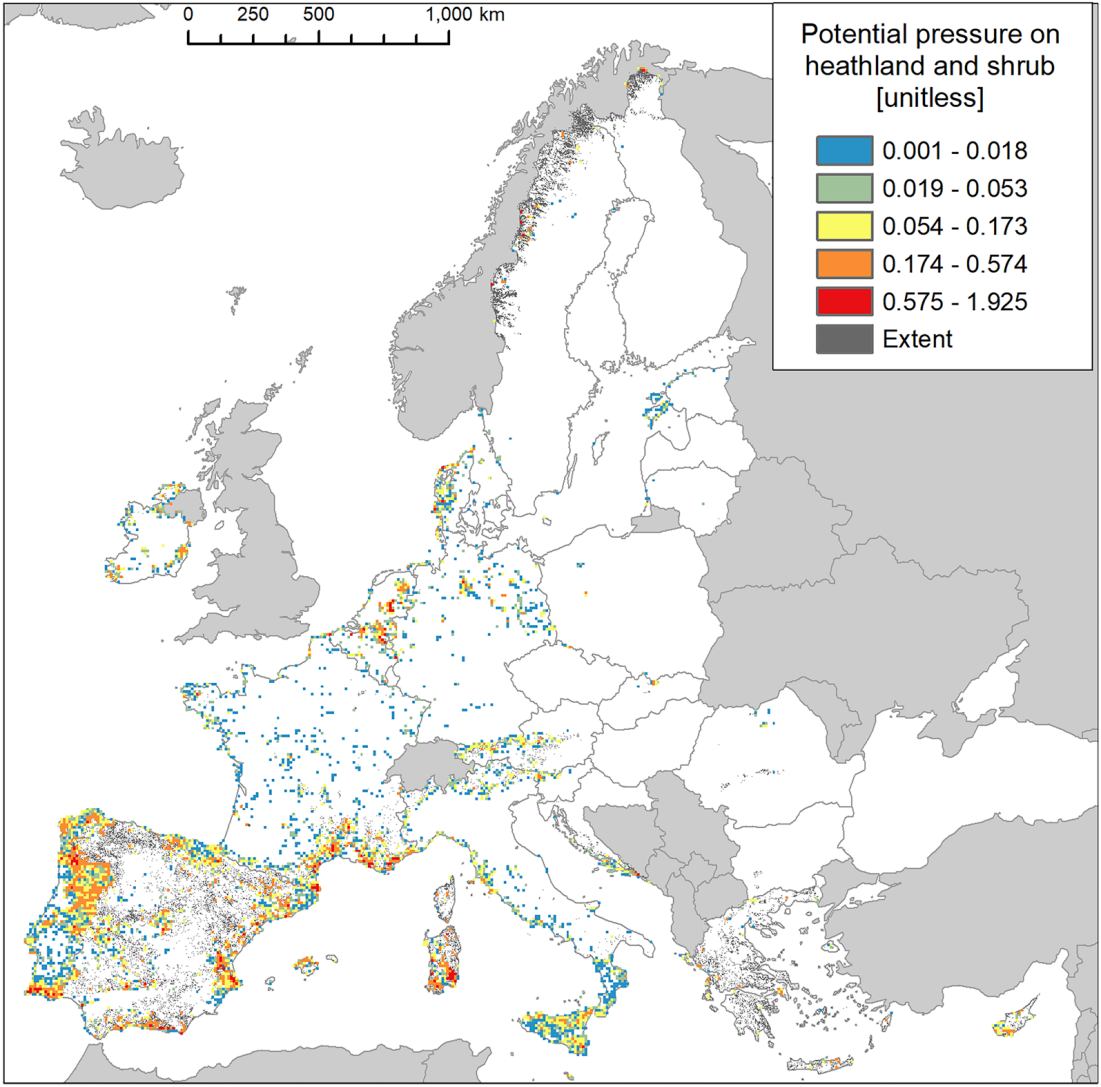
Potential pressure by IAS on heathland and shrub, mapped on the 100-km^2^ reference squares. Extent indicates areas of heathland and shrub without reporting of IAS (dark grey). Countries beyond the geographic scope of the study are in light grey. See main text for additional details.

Potential pressure in the highest end was recorded across Portugal, the Mediterranean side of Spain and France, the Sardinia Island, Belgium, and Netherlands (Fig. 14). The number of reported IAS, the relative extent of invaded areas, and the percent of invasion by each IAS are shown in the “Supplementary Information” (Fig. S6, Tables S13 and S14).

### Sparsely vegetated land ecosystems

**Figure 15:**
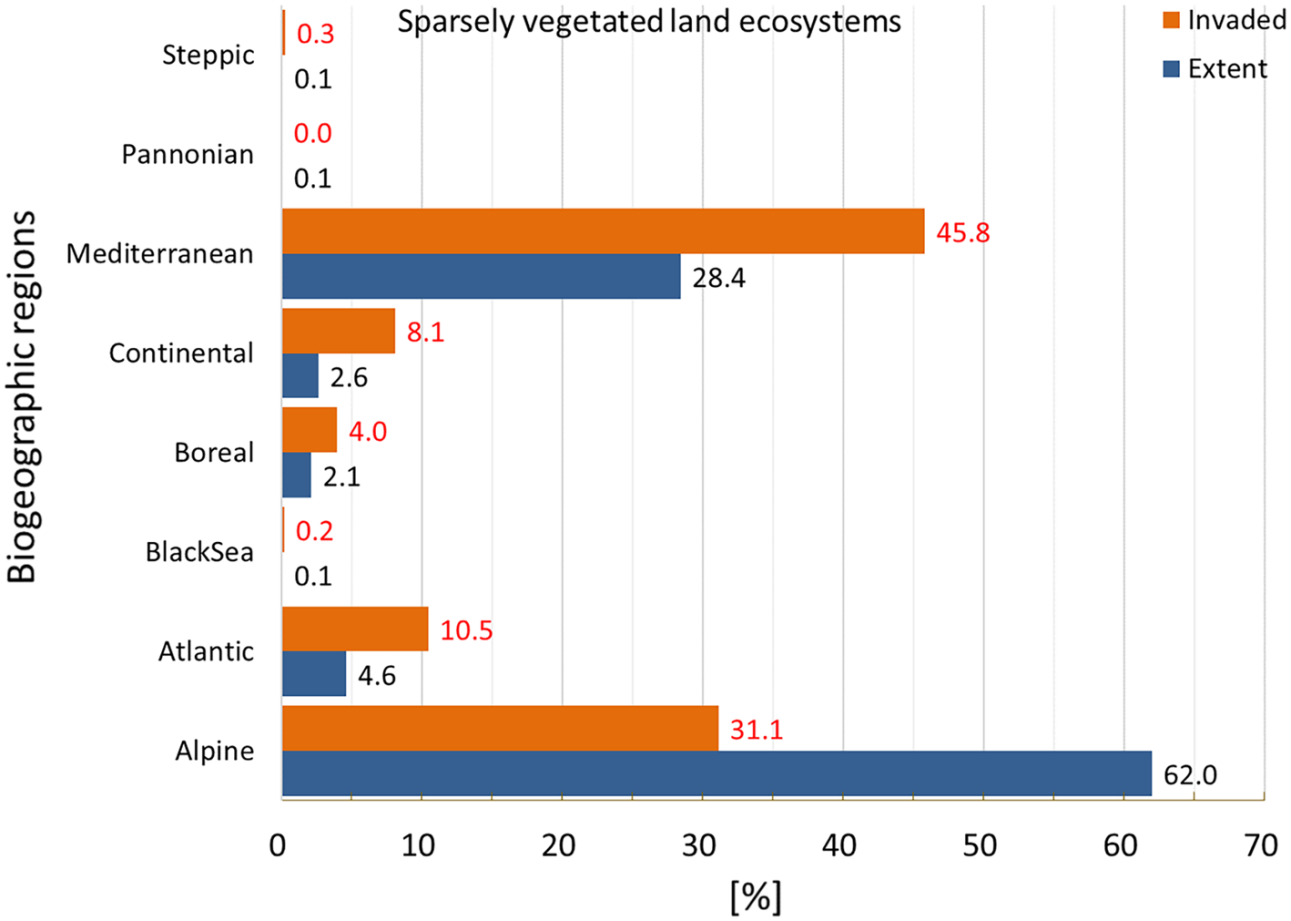
Biogeographic characterisation of sparsely vegetated land: extent versus invasion. Label on bars indicate the correspondent area as percent of the total extent (in blue) or as percent of the total invaded area (in orange).

Invasion was present just above 19% of sparsely vegetated land, ranking this ecosystem as the least invaded (Fig. 2). Except for Pannonian and Alpine regions, all other regions were disproportionally invaded, with Steppic and Continental regions ranking at the top (Fig. 15). The statistics showed large variation in potential pressure around the mean value, with the second highest coefficient of variation across terrestrial ecosystems, after urban ecosystems and a median ca. 61% lower than the mean (mean ± SD: 0.052 ± 0.096; CV: 184.7%; median: 0.020; Suppl. Table S3).

**Figure 16:**
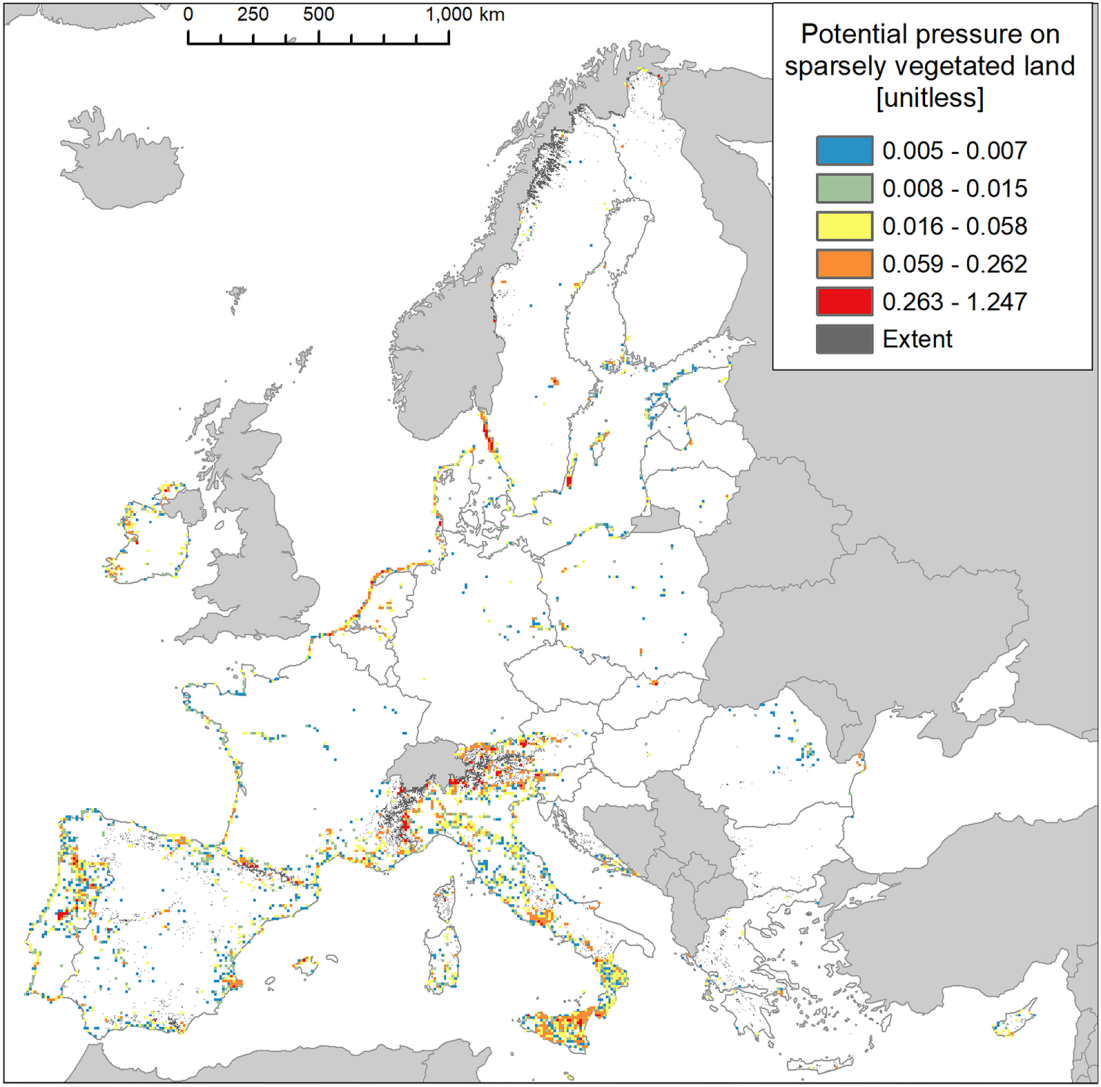
Potential pressure by IAS on sparsely vegetated land, mapped on the 100-km^2^ reference squares. Extent indicates areas of sparsely vegetated land without reporting of IAS (dark grey). Countries beyond the geographic scope of the study are in light grey. See main text for additional details.

Hot spots of high values potential pressure in the highest end were recorded in Portugal, the Alpine region, the island of Sicily (Italy), and Sweden, particularly along the western coast (Fig. 16). Taken together with the biogeographical patterns, these results suggest that invasion across the Alpine region is less widespread but characterised by hot spots of high values potential pressure. The number of reported IAS, the relative extent of invaded areas, and the percent of invasion by each IAS are shown in the “Supplementary Information” (Fig. S7, Tables S15 and S16).

### Freshwater ecosystems

**Figure 17:**
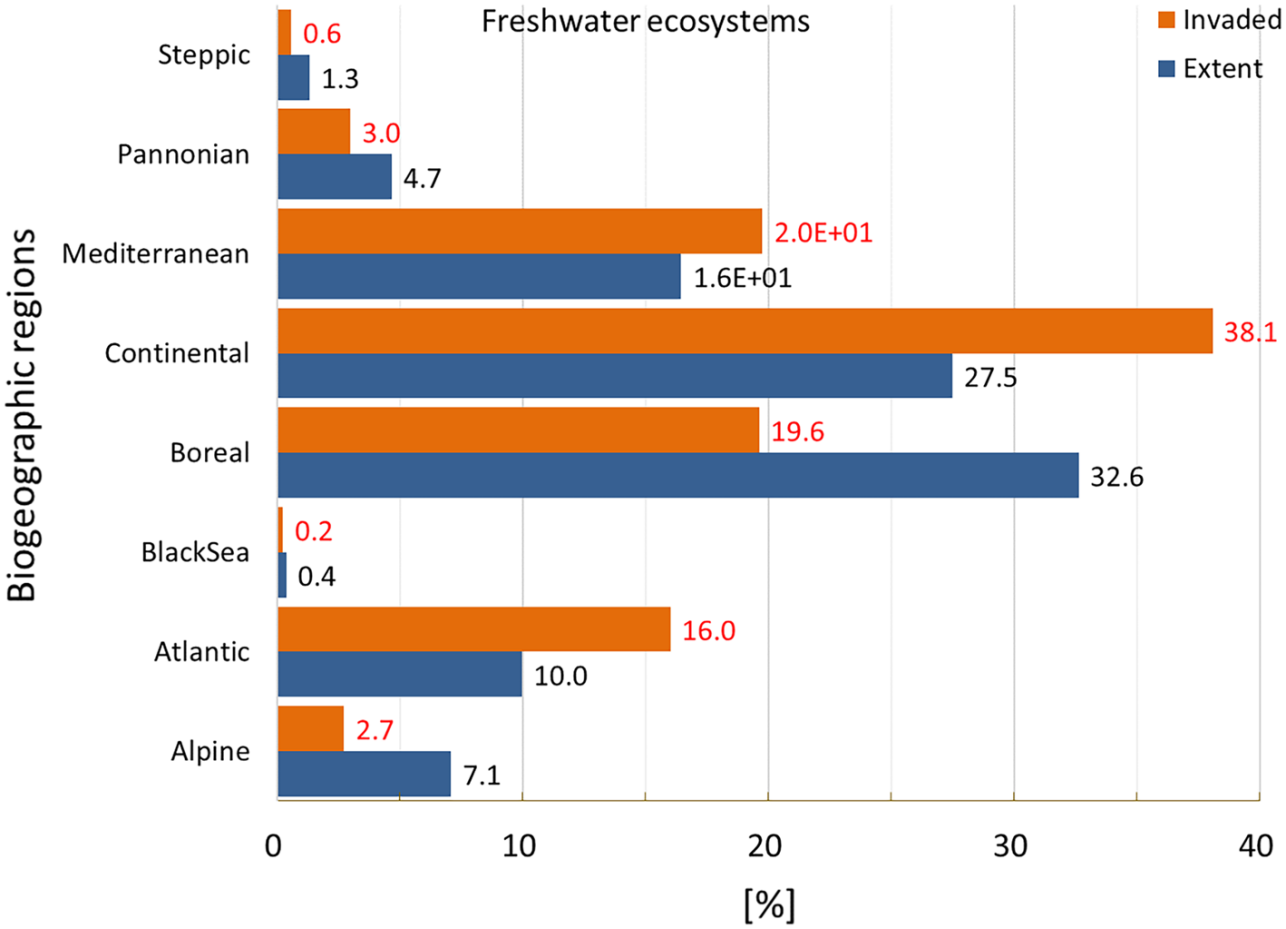
Biogeographic characterisation of freshwater ecosystems: extent versus invasion. Label on bars indicate the correspondent area as percent of the total extent (in blue) or as percent of the total invaded area (in orange). See main text for additional details.

Invasion was recorded just above 52% of freshwater ecosystems, placing this ecosystem type as the second most invaded after urban ecosystems (Fig. 2). The presence of IAS in freshwater ecosystems was disproportionally greater in the Atlantic region, followed by Continental and Mediterranean regions (Fig. 17). The statistics showed large variation in potential pressure around the mean value, with the second highest coefficient of variation across all ecosystems after urban ecosystems, and a median ca. 69% lower than the mean (mean ± SD: 0.204 ± 0.457; CV: 223.6%; median: 0.063, Suppl. Table S3).

**Figure 18:**
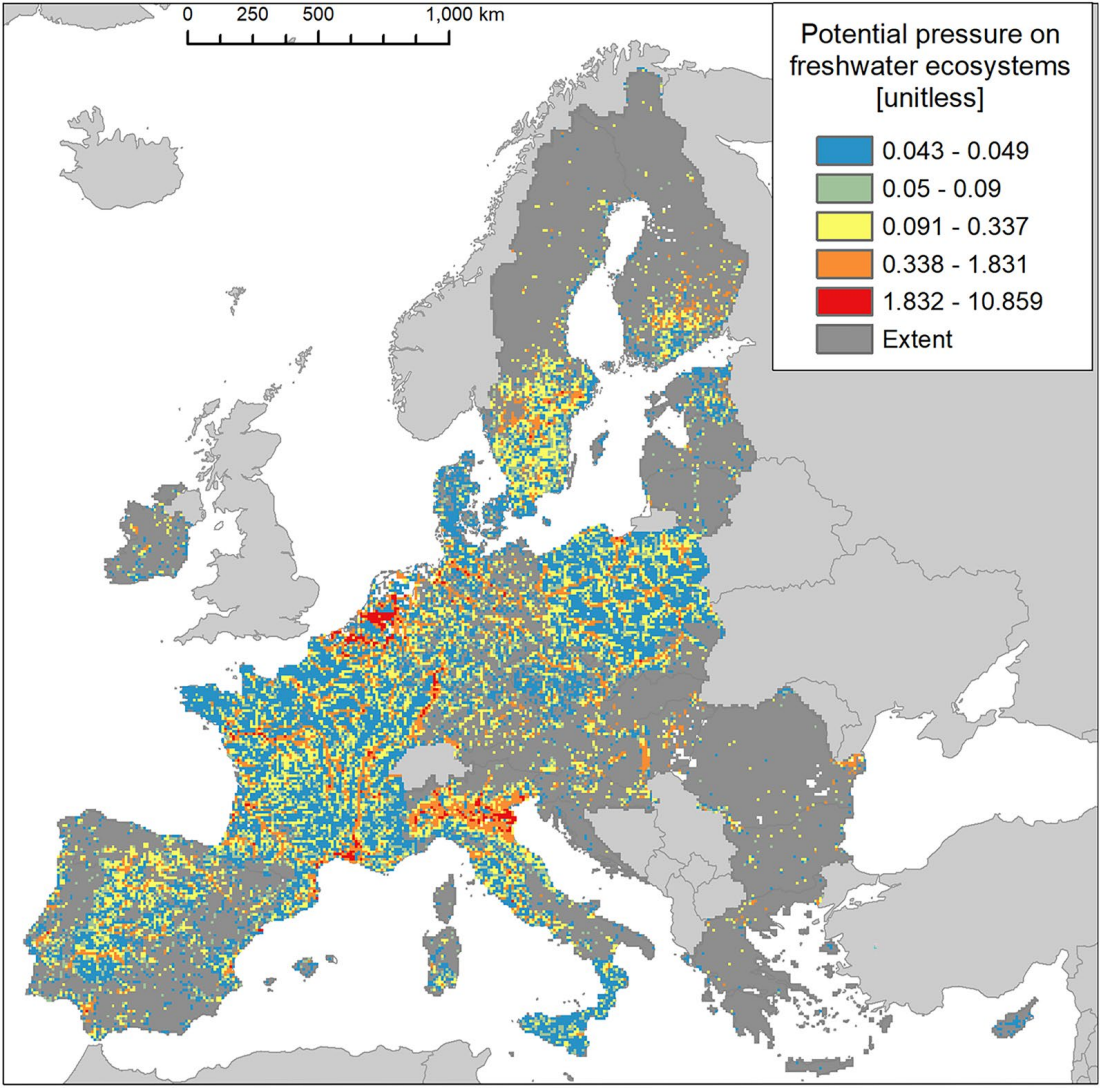
Potential pressure by IAS on freshwater ecosystems, mapped on the 100-km^2^ reference squares. Extent indicates areas of freshwater ecosystems without reporting of IAS (dark grey). Countries beyond the geographic scope of the study are in light grey. See main text for additional details.

Potential pressure across freshwater ecosystems reached almost 10.9 (Fig. 18), the highest value recorded across all ecosystem types. Most of the values, however, were distributed in the lowest range of potential pressure (Fig. 3). Potential pressure in the highest end were recorded on the delta of the River Rhone (the Camargue region in the southern part of France), along the course and delta of the River Po and of some of its tributaries, in Northern Italy. Widespread potential pressure was also recorded in the northern part of Flanders, Belgium, and in the Noord-Brabant and Gelderland regions in the Netherlands. Several other hotspots of high potential pressure were also recorded along the River Rhine, particularly as it flows along the Franco-German border and western Germany (Fig. 18). The number of reported IAS, the relative extent of invaded areas, and the percent of invasion by each IAS are shown in the “Supplementary Information” (Fig. S8, Tables S17 and S18).

## Methods

The potential pressure of 66 invasive alien species of Union concern on terrestrial and freshwater ecosystems across the European Union (EU, 27 members in 2022) was quantified through an indicator that accounted for the number of IAS present in a given area and the extent of the ecosystem(s) affected. The geographic scope of the assessment excluded the outermost regions of the EU. To quantify the potential pressure, we considered these elements:

- The distribution records of the IAS of Union concern available on the European Invasive Species Information Network (EASIN https://easin.jrc.ec.europa.eu/easin) at the 10-km spatial resolution of the European Environment Agency (EEA) reference grid (https://www.eea.europa.eu/data-and-maps/data/eea-reference-grids-2). Distributional data were available for all species with the exception of: *Ehrharta calycina*, *Lespedeza cuneata* (*Lespedeza juncea var. sericea*), *Microstegium vimineum* and *Triadica sebifera* (*Sapium sebiferum*). None of these four species had been reported as present in the EU, at the time of this study.
- The potential pressure caused by IAS on each ecosystem. This information was based on the traits of the IAS, obtained from Tsiamis et al.^24^ for the first 37 species listed as of Union concern, from Tsiamis et al.^25^ for the 12 species added in 2017, and from Tsiamis et al.^26^ for the 17 species added to the list in 2019. Specifically, we translated known impacts caused by IAS on invaded ecosystems reported in the cited documents to an indication of potential pressure on the relevant ecosystem (Suppl. Tables S1 and S2).
- The distribution and extent of ecosystem types. For terrestrial ecosystems, we adopted the typology developed by Maes et al.^23^ for mapping and assessing ecosystems and their services (MAES) across the European Union. We applied this typology to the 2018 version of CORINE land cover classification system (100-m spatial resolution) and linked it to the relevant potential pressure (Suppl. Table S4). Freshwater ecosystems comprised rivers, lakes, and riparian land. The extent of rivers and lakes was derived from combining information of the hydrological network as represented by Catchment Characterisation Model (CCM)^27^, but removing brackish or salty water bodies as classified by CORINE 2012 into classes 37–39 and 42–44. The area of aquatic ecosystems (river and lakes, km^2^) was quantified by buffering CCM river segments with a 5-m buffer on each side, i.e., assuming a constant river cross section of 10 m everywhere. Rivers and lakes were then merged with riparian land^28^. The dataset is available in Grizzetti et al.^29^.

For each 100-km^2^ area (grid cell) where an IAS was recorded, we quantified the potential pressure as the cumulative extent of all ecosystems potentially affected by the presence of the IAS, based on the species’ traits information listed above. Our additive model, therefore, is a conservative approach based on the CIMPAL Index proposed by Katsanevakis et al.^30^. Unlike the CIMPAL index, our approach assesses the potential pressure in a binary way: evidence of pressure or absence of evidence.

Equation 1 shows the mathematical components of the Index of cumulative potential pressure (I_c_):

1 $${I}_{c }=\sum_{s=1}^{S}\sum_{e=1}^{E}{O}_{s}{H}_{e}{w}_{s,e}$$

where: *I*_*c*_ is the cumulative potential pressure for grid cell c (0–S); *s* is the invasive alien species; *e* is the ecosystem type; *O*_*s*_ is the occurrence of species in grid cell c (0, 1); *H*_*e*_ is the share of ecosystem type e within grid cell c (0–1); *w*_*s,e*_ is the evidence of pressure of species s on the ecosystem type e (0, 1).

It follows that the cumulative potential pressure for a grid cell and the ecosystems within it ranges from zero to the total number of IAS present in that cell.

We present the results across all ecosystems and for each ecosystem separately, together with summary statistics. Statistics were based only on the invaded 100-km^2^ reference grid cells, i.e., where potential pressure was greater than zero. Potential pressures were rounded to the third decimal digits during reporting of the results; hence values smaller than 0.001 were not reported. Such values might be produced, for instance, for patches of ecosystems of a few hectares (< 5 ha), invaded by one IAS. Given the broad variation in ecosystem extents, we also computed the coefficient of variation (CV).

The biogeographical pattern of invasion for each ecosystem was derived from the EEA biogeographical regions dataset (https://www.eea.europa.eu/data-and-maps/data/biogeographical-regions-europe-3). For each ecosystem, the relative extent of each bioregion within the EU, without its outermost regions (Suppl. Fig. S1), was compared to the relative extent invaded by IAS.

## Discussion

We assessed the potential pressure caused by 66 IAS of EU concern on the ecosystems they invade, looking simultaneously at the distribution of IAS and the extent of the ecosystems they can negatively affect. This approach allowed us to assess the distribution as well as the magnitude of potential pressure across the EU.

The biogeographical pattern of invasion across EU revealed a disproportionally greater invasion in the Atlantic region, followed by Continental and Mediterranean regions. Most IAS of EU concern have been introduced and have spread across north-western EU countries, while their presence is more limited in southern EU countries. This might be related to historical reasons: the majority of first introduction events of IAS of Union concern, in fact, took place in France and in Ireland^24,25^. Atlantic and Continental regions were also highlighted as hotspots of invasive alien terrestrial vertebrates in Europe (IATV) in a recent work by Polaina et al.^31^ using records from GBIF, with more than two thirds of them belonging to the list of Union concern. Polaina et al.^31^ found that most of the southern and eastern EU countries are areas where additional data are most needed. Similar pattern is reported for first introduction events of terrestrial plants, with northwestern European countries acting as the main gateway areas of alien plants into Europe^32^, while central/western countries were the main gateways for freshwater alien species^33^. High level of invasion by neophytes across Atlantic and Continental regions in Europe, and low levels across Boreal regions were also predicted by Chytrý et al.^34^ from over 50,000 vegetation plots representative of broad and contrasting European climate regions.

The analysis of invasion across ecosystems revealed that urban and freshwater ecosystems were the most invaded (nearly 68% and ca. 52% of their extent respectively), followed by forest and woodland (nearly 44%). To notice, the EU member states’ reporting of IAS in habitats protected by the Habitats Directive^35^ , showed that IAS of Union concern are most often detected in coastal habitats, followed by forest and freshwater habitats^36^. Urban ecosystems include cities and their surroundings; they are socio-ecological systems where most people live. They are peculiar ecosystem types because they are predominantly artificial, but they include, in different proportions, several other ecosystems (forests, lakes, rivers and agricultural areas can all be part of urban fringe) and they are strongly influenced by human activities. These characteristics can contribute to explain the high percentage of invasion reported in urban ecosystems (also predicted by Chytrý et al.^34^). European cities are remarkably diverse in terms of land composition, policymaking, territorial development, urbanization, and demographic transition: this diversity can contribute to explain the high variability observed in the shares of invaded area, which is the highest one across the ecosystem types we have considered. Despite these differences, however, urbanized areas share common problems, such as noise and air pollution, flood risk, heat island effect, scarce presence of green spaces. Therefore, urban environmental policies are gaining importance in both the EU and international agenda (e.g., Goal 11 of the Sustainable Development Goals). While it has been argued that urban areas could be managed to become colonies for endangered non-native species^37^, evidence shows that urban areas can also provide habitats to native biodiversity (and in some cases also act as hotspots, e.g.^38^), and that outdoor urban areas might contribute to ensure food security^39^. This body of evidence encourages setting up management actions to lower the negative impacts caused by IAS on native biodiversity^40^, man-made features and agriculture^41,42^.

Our assessment reveals freshwaters as the second most invaded ecosystem, with IAS reported in ca. 52% of their area. A recent analysis of the distribution and potential impacts of IAS, including those of EU concern, in European catchments, found a significant relation between the CIMPAL index^30^ and the ecological status of freshwater according to the EU Water Framework Directive^43^, with increasing values of CIMPAL associated to poorer ecological status in eight European countries^44^.

We found that the average potential pressure of IAS was greater across cropland and forests, which were characterized also by the lowest coefficient of variation. Previous work not restricted to the species on the Union list found the proportion of neophytes to all plant species among the highest ones in agricultural areas^34^. Taken together, these findings raise concerns on the potential consequences on ecosystem functioning. Documented impacts of IAS in cropland, in fact, include spreading of pests and pathogens and disturbance of plant-pollinators networks, with certain invasive alien plant species attracting pollinators more than the native ones^45^. Additional impacts include adverse environmental as well as socio-economic impacts, by disturbing native biodiversity, affecting ecosystem functioning and therefore the capacity of ecosystems to provide services. *Vespa velutina nigrithorax*, for instance, is a known predator of pollinating insects; its spread may cause economic losses related to the reduction of fruit production in response to predation of pollinating insects^46^. *Impatiens glandulifera* affects soil microbial attributes through disruption of mycorrhizal association^47^; *Ailanthus altissima* can cause allergic reactions in humans^48^.

Sparsely vegetated land, and heathland and shrub, were the ecosystems with lowest level of invasion (and potential pressure). Similar patterns were also reported for invasion by neophytes^34^.

In grassland ecosystems, we found disproportionally greater invasion in Continental and Atlantic regions. A wide range of European grasslands studied from over 90,000 vegetation plots, revealed greater invasion of neophytes in Continental and Boreal regions^49^. Confounding factors potentially explaining this difference are the restricted number of invasive alien species we have considered, their coarse grain mapping, and potential gaps in their reporting.

The records of IAS used for this study were available at the 10-km spatial resolution; hence, the actual presence of the species at finer resolution could not be evaluated, nor was it possible to ascertain if that species actually exerted pressure on all susceptible ecosystems in a given 100-km^2^ cell. For this reason, throughout our assessment, we used ‘potential pressure’ rather than ‘pressure’. For national or supra-national analyses, the spatial resolution of the data used in this study might be suitable; but to identify areas where measures should be prioritized, a finer spatial resolution is needed. In addition, our assessment did not comprise many IAS that, while also causing severe negative impacts, are not included in the Union list. Examples are *Carpobrotus acinaciformis*, *C. edulis*, *Robinia pseudoacacia*, which score among the 100 worst alien species in Europe based on 12 categories of impacts^50^, but are not on the Union list. Evidence and severity of impacts, in fact, are not the only criteria for inclusion on the EU regulated list: management costs, and effective prevention and mitigation of the impacts, are also criteria considered for inclusion in the Union list. The results of our assessment, therefore, might support actions towards the IAS considered, but patterns might not necessarily reflect what obtained when also adding IAS that are not on the Union list.

Information about IAS was limited to presence-only data, preventing us from considering species abundance, which may vary considerably across the distributional range. As a conservative application of the CIMPAL Index proposed by Katsanevakis et al.^30^, we did not weigh the magnitude of pressure based on the known impact caused by the IAS, nor did we distinguish between impact types, because all the IAS of EU concern pose significant threat to the ecosystems they invade. We are aware, however, that their actual impacts are not necessarily similar, and that these differences may influence management priorities and types of intervention. The impacts of IAS on biodiversity and ecosystem services are complex and often take substantial time to become evident: the effects of an IAS cannot be predicted straightforwardly by looking at the ecology of the species in its native range^51,52^. Furthermore, the impacts of an IAS might be influenced by local environmental conditions, susceptibility of the ecosystem, current trend of climatic conditions, and socio-economic aspects. Hence, currently, we cannot translate the presence of an IAS into a level of ecosystem degradation. In general, understanding and quantifying IAS impacts, not only from the research-based evidence perspective, but also from the methodological point of view of achieving standardized practices, remains a challenge^53^. Moreover, the consequences caused by the presence of several IAS in the same area, could be more severe than the sum of each species impact^44^. Advancing on these aspects is crucial to support management, prioritization, adoption, and implementation of effective intervention measures: our findings in fact, document for the first time to our knowledge, that invasive alien species could exert high pressure on terrestrial and freshwater ecosystems across large areas of Europe.

## Supplementary Information

Supplementary Information.

## Data Availability

The datasets analyzed during the current study are available from the corresponding author on reasonable request, as well as from the public repository cited in the ‘Methods’ (EASIN European Invasive Species Information Network: https://easin.jrc.ec.europa.eu/easin).
