# Supplementary material for: Invasive alien species of policy concerns show widespread patterns of invasion and potential pressure across European ecosystems
Source: Sci Rep. 2023 May 19;13:8124. doi: 10.1038/s41598-023-32993-8 (PMC10199087; doi:10.1038/s41598-023-32993-8)
Supplement: Supplementary file 1 — Supplementary Information. [file 41598_2023_32993_MOESM1_ESM.pdf]

Supplementary Information

Contents

Invasive alien species of Union Concern (2019) 1
Summary statistics 4
Ecosystem types and links with potential pressure 5
Biogeographical regions 7
Patterns of invasion 8
Urban ecosystems 8
Cropland ecosystems 10
Grassland ecosystems 12
Forest and woodland ecosystems 14
Heathland and shrub ecosystems 16
Sparsely vegetated land ecosystems 18
Freshwater ecosystems 20
Cited bibliography 21

Invasive alien species of Union Concern (2019)

Table S 1: The 36 plant species on the list of Invasive Alien Species (IAS) of Union Concern [1], and their potential pressure (1,0) on artificial areas and macro-categories of ecosystems. 1 = evidence of pressure; 0 = no evidence.

Species name	English name	Environmental macro-categories affected by IAS pressure			
		Man-made structures and urban areas	Cropland	Forest and semi-natural areas	Freshwater ecosystems
Acacia saligna (A. cyanophylla)	Golden wreath wattle	0	1	1	0
Ailanthus altissima	Tree of heaven	1	0	1	0
Alternanthera philoxeroides	Alligator weed	1	1	1	1
Andropogon virginicus	Broomsedge bluestem	0	0	1	0
Asclepias syriaca	Common milkweed	1	1	1	0
Baccharis halimifolia	Eastern baccharis	1	0	1	0
Cabomba caroliniana	Fanwort	0	1	0	1
Cardiospermum grandiflorum	Balloon vine	0	0	1	0
Cortaderia jubata	Purple pampas grass	1	1	1	0
Ehrharta calycina	Perrenial veldt grass	0	0	1	0
Eichhornia crassipes	Water hyacinth	1	0	0	1
Elodea nuttallii	Nuttall's waterweed	1	0	0	1
Gunnera tinctoria	Chilean rhubarb	0	0	1	0
Gymnocoronis spilanthoides	Senegal tea plant	1	0	1	1
Heracleum mantegazzianum	Giant hogweed	1	0	1	0
Heracleum persicum	Persian hogweed	1	0	1	0
Heracleum sosnowskyi	Sosnowsky's hogweed	1	0	1	0
Humulus scandens	Japanese hop	1	0	1	0
Hydrocotyle ranunculoides	Floating pennywort	1	0	0	1
Impatiens glandulifera	Himalayan balsam	0	0	1	0
Lagarosiphon major	Curly waterweed	1	0	0	1
Lespedeza cuneata (L. juncea var. sericea)	Chinese bushclover	0	0	1	0
Ludwigia grandiflora	Water-primrose	1	0	0	1
Ludwigia peploides	Floating primrose-willow	1	0	0	1
Lygodium japonicum	Vine-like fern	0	1	1	0
Lysichiton americanus	American skunk cabbage	0	0	1	0
Microstegium vimineum	Japanese stiltgrass	1	0	1	0

Continued on next page

Continued from previous page *(Table S 1: The 36 plant species on the list of Invasive Alien Species (IAS) of Union Concern[1], and their potential pressure (1,0) on artificial areas and macro-categories of ecosystems. 1 = evidence of pressure; 0 = no evidence.*

Species name	English name	Environmental macro-categories affected by IAS pressure			
		Man-made structures and urban areas	Cropland	Forest and semi-natural areas	Freshwater ecosystems
Myriophyllum aquaticum	Parrot's feather	1	0	0	1
Myriophyllum heterophyllum	Broadleaf watermilfoil	1	0	0	1
Parthenium hysterophorus	Whitetop weed	1	1	1	0
Pennisetum setaceum	Crimson fountaingrass	0	0	1	0
Persicaria perfoliata	Asiatic tearthumb	0	1	1	0
Prosopis juliflora	Mesquite	1	0	1	0
Pueraria montana var. lobata	Kudzu vine	1	1	1	0
Salvinia molesta (S. adnata)	Salvinia moss	1	0	0	1
Triadica sebifera (Sapium sebiferum)	Chinese tallow	1	0	1	0

Table S 2: The 30 animal species on the list of Invasive Union Concern [1], and their potential pressure (1,0) on artificial areas and macro-categories of ecosystems. 1 = evidence of pressure; 0 = no evidence.

Species name	English name	Environmental macro-categories affected by IAS pressure			
		Man-made structures and urban areas	Cropland	Forest and semi-natural areas	Freshwater ecosystems
Acridotheres tristis	Common myna	1	0	1	0
Alopochen aegyptiacus	Egyptian goose	0	1	1	1
Arthurdendyus triangulates	New Zealand flatworm	0	1	1	0
Callosciurus erythraeus	Pallas' squirrel	0	0	1	0
Corvus splendens	Indian house crow	1	1	1	0
Eriocheir sinensis	Chinese mittencrab	0	0	0	1
Herpestes javanicus	Small Asian mongoose	1	1	1	0
Lepomis gibbosus	Pumpkinseed	0	0	0	1
Lithobates catesbeianus	American bullfrog	0	0	0	1
Muntiacus reevesi	Muntjac deer	0	1	1	0
Myocastor coypus	Coypu	1	1	1	1
Nasua nasua	Coati	0	0	1	0
Nyctereutes procyonoides	Raccoon dog	1	0	1	0
Ondatra zibethicus	Muskrat	1	1	1	1
Orconectes limosus	Spiny-cheek crayfish	0	0	0	1
Orconectes virilis	Virile crayfish	0	0	0	1
Oxyura jamaicensis	Ruddy duck	0	0	1	1
Pacifastacus leniusculus	Signal crayfish	0	0	0	1
Perccottus glenii	Amur sleeper	0	0	0	1
Plotosus lineatus	Striped eel catfish	0	0	0	1
Procambarus clarkii	Red swamp crayfish	1	1	0	1
Procambarus fallax f. virginalis	Marbled crayfish	0	0	0	1
Procyon lotor	Raccoon	1	1	1	0
Pseudorasbora parva	Stone moroko	0	0	0	1
Sciurus carolinensis	Grey squirrel	0	0	1	0
Sciurus niger	Fox squirrel	1	0	1	0
Tamias sibiricus	Siberian chipmunk	0	1	1	0
Threskiornis aethiopicus	Sacred ibis	0	0	1	1
Trachemys scripta	Pond slider	0	0	0	1
Vespa velutina nigrithorax	Asian hornet	1	1	1	0

Summary statistics

Table S 3: Summary statistics of the cumulative potential pressure by IAS across ecosystem types. Freshwater is an aggregated ecosystem type, which includes wetlands, rivers and lakes. Statistics were computed for invaded areas only, with each area corresponding to an extent of ecosystem within a 100-km² reference grid cell. The number of invaded cells is indicated by N, which represents therefore the simple size for computing summary statistics. Potential pressure was rounded to the 3rd decimal digit during reporting, hence 0.001 is the minimum reported value. Given the differences in sample size, we also report the coefficient of variation (CV %).

Ecosystem type	Invaded area (%)	N	Min	Max	Arithmetic mean ± SD	CV (%)	Median
Urban	67.7	18471	0.001	8.717	0.172 ± 0.449	261.0	0.050
Freshwater	52.3	20023	0.001	10.859	0.204 ± 0.456	223.6	0.063
Forest & woodland	44.0	20712	0.001	6.268	0.541 ± 0.528	97.6	0.405
Cropland	42.9	14785	0.001	4.82	0.656 ± 0.544	82.9	0.554
Grassland	38.9	14084	0.001	3.994	0.190 ± 0.294	154.8	0.084
Heathland & shrub	23.4	4757	0.001	1.925	0.111 ± 0.165	149.0	0.046
Sparsely vegetated land	20.6	3450	0.001	1.316	0.056 ± 0.108	191.2	0.020

Ecosystem types and links with potential pressure

Table S 4: Cross-tabulation of CORINE LC classes' level 3, MAES ecosystem types' level 2, and macro-category of ecosystems adopted to identify the presence of potential pressure.

CORINE LC level 3	MAES level 2 ecosystem type	Macro-category of ecosystems
Continuous urban fabric	Urban	Artificial
Discontinuous urban fabric		
Industrial or commercial units		
Road and rail networks and associated land		
Port areas		
Airports		
Mineral extraction sites		
Dump sites		
Construction sites		
Green urban areas		
Sport and leisure facilities		
Non-irrigated arable land	Cropland	Agriculture
Permanently irrigated land		
Rice fields		
Vineyards		
Fruit trees and berry plantations		
Olive groves		
Pastures	Grassland	
Annual crops associated with permanent crops	Cropland	
Complex cultivation patterns		
Land principally occupied by agriculture with significant areas of natural vegetation		
Agro-forestry areas		

Continued on next page

Continued from previous page (Table S 4: Cross-tabulation of CORINE LC classes' level 3, MAES ecosystem types' level 2, and macro-category of ecosystems adopted to identify the presence of pressure).

**CORINE LC level 3**	**MAES level 2 ecosystem type**	**Macro-category of ecosystems**
Broad-leaved forest	Woodland and forest ('Forest')	Forest & Semi-natural
Coniferous forest		
Mixed forest		
Natural grasslands	Grassland	
Moors and heathland	Heathland and shrub	
Sclerophyllous vegetation		
Transitional woodland-shrub	Woodland and forest ('Forest')	
Beaches dunes sands	Sparsely vegetated land	
Bare rocks		
Sparsely vegetated areas		
Burnt areas		
Glaciers and perpetual snow		
Inland marshes	Wetlands	Freshwater
Peat bogs		
Salt marshes	Marine inlets and transitional waters	Excluded
Salines		
Intertidal flats		
Water courses	Rivers and lakes	Freshwater
Water bodies		
Coastal lagoons	Excluded	Excluded
Estuaries		
Sea and ocean		

Biogeographical regions

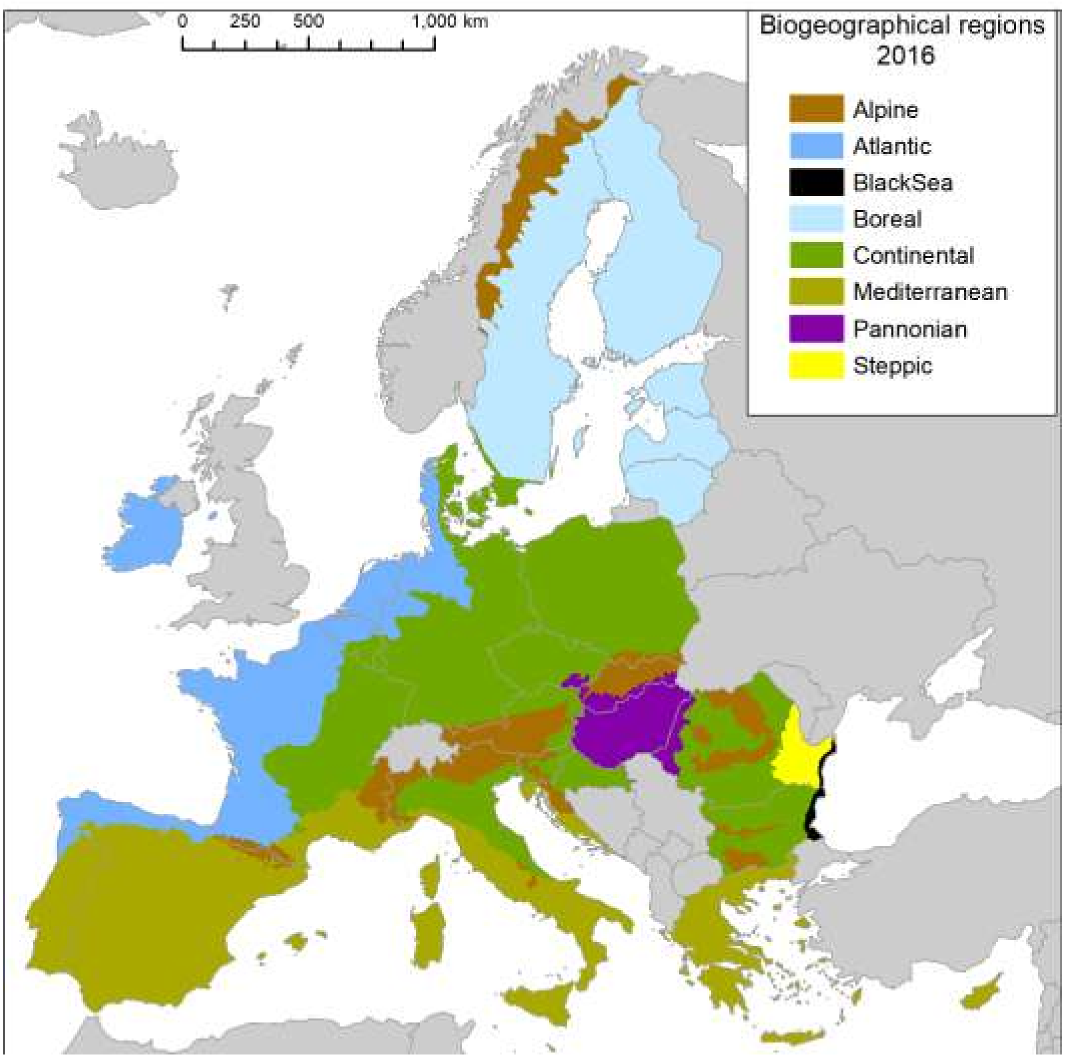

Figure S 1: Biogeographical regions (2016) across the European Union (2022) without its outermost regions. Source of information: European Environment Agency (https://www.eea.europa.eu/data-and-maps/data/biogeographical-regions-europe-3)

.

Patterns of invasion

Urban ecosystems

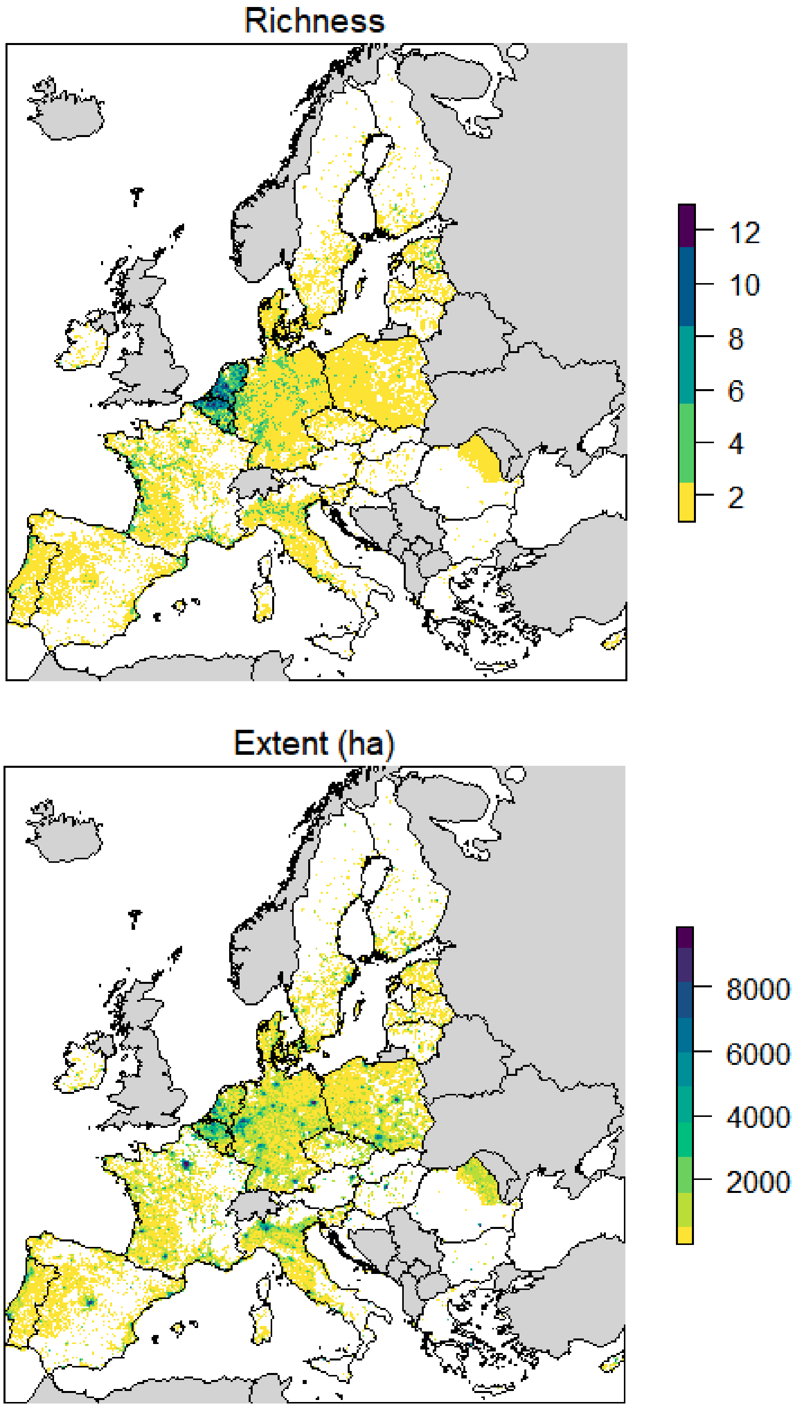

Figure S 2: Number of reported IAS (top) and extent of invaded areas (bottom) across urban ecosystems. Extent refers to invaded hectares within the 100-km² grid cell. Countries beyond the geographic scope of the study are in light grey.

Table S 5: Relative extent (%) of invaded urban ecosystems by animal IAS, in descending order.

Species	*Extent (%)*
Procyon lotor	14.77
Myocastor coypus	12.08
Ondatra zibethicus	8.19
Procambarus clarkii	6.91
Vespa velutina nigrithorax	5.21
Nyctereutes procyonoides	2.12
Acridotheres tristis	0.41
Sciurus niger	0.19
Corvus splendens	0.12
Herpestes javanicus	0.02

Table S 6: Relative extent (%) of invaded urban ecosystems by plant IAS, in descending order.

Species	*Extent (%)*
Ailanthus altissima	10.04
Heracleum mantegazzianum	9.74
Heracleum sosnowskyi	5.94
Elodea nuttallii	4.70
Myriophyllum aquaticum	4.54
Ludwigia grandiflora	4.19
Hydrocotyle ranunculoides	3.10
Baccharis halimifolia	1.72
Ludwigia peploides	1.42
Lagarosiphon major	1.28
Eichhornia crassipes	0.98
Asclepias syriaca	0.85
Myriophyllum heterophyllum	0.53
Heracleum persicum	0.44
Salvinia molesta	0.19
Pueraria montana var. lobata	0.15
Humulus scandens	0.10
Alternanthera philoxeroides	0.03
Cortaderia jubata	0.02
Gymnocoronis spilanthoides	0.01
Parthenium hysterophorus	0.01
Prosopis juliflora	< 0.01

Cropland ecosystems

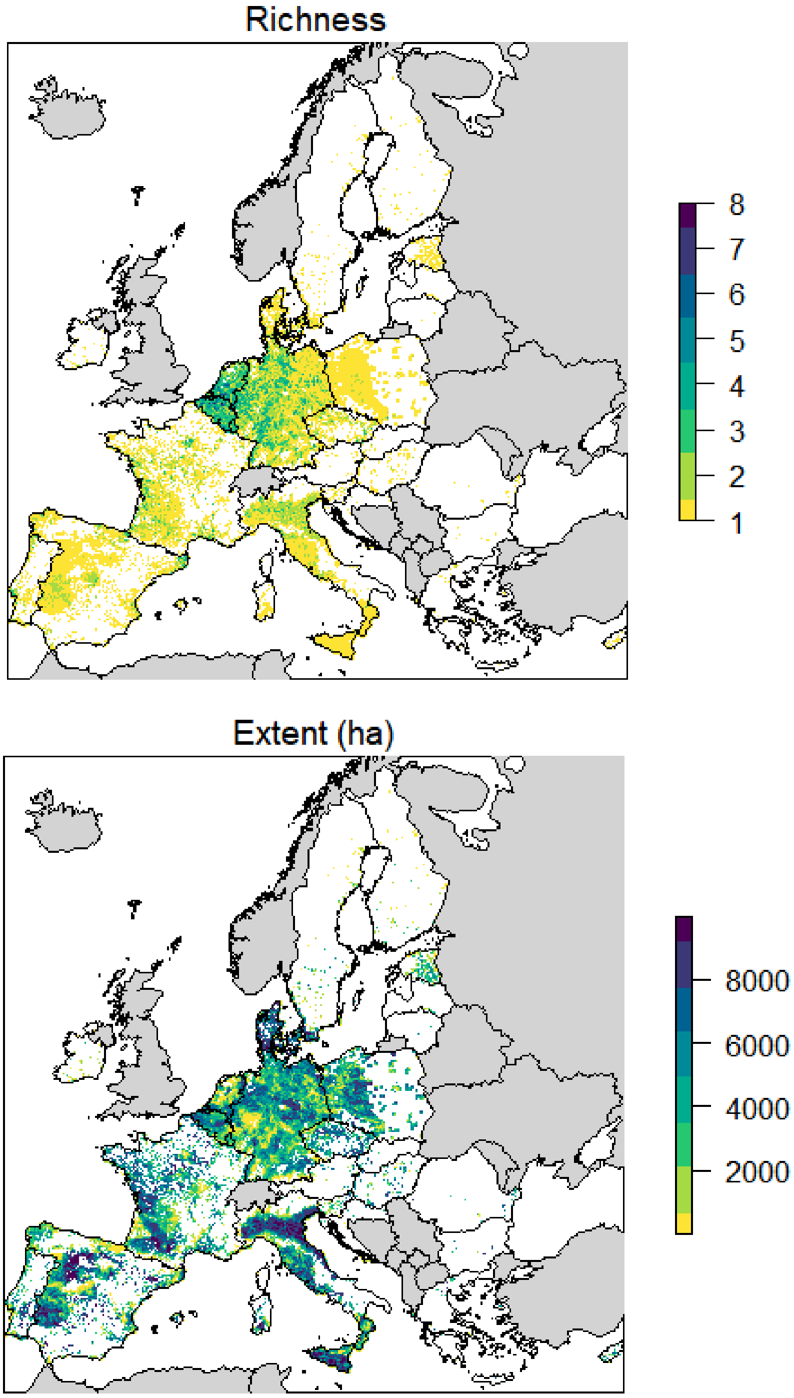

Figure S 3: Number of reported IAS (top) and extent of invaded areas (bottom) across cropland ecosystems. Extent refers to invaded hectares within the 100-km^2 grid cell. Countries beyond the geographic scope of the study are in light grey.

Table S 7: Relative extent (%) of invaded cropland ecosystems by animal IAS, in descending order.

Species	*Extent (%)*
Procyon lotor	21.93
Alopochen aegyptiacus	21.05
Myocastor coypus	19.80
Procambarus clarkii	17.30
Vespa velutina nigrithorax	9.64
Ondatra zibethicus	7.15
Muntiacus reevesi	0.50
Tamias sibiricus	0.37
Corvus splendens	0.08
Herpestes javanicus	0.04
Arthurdendyus triangulatus	0.02

Table S 8: Relative extent (%) of invaded cropland ecosystems by plant IAS, in descending order.

Species	*Extent (%)*
Asclepias syriaca	0.85
Acacia saligna	0.72
Cabomba caroliniana	0.42
Pueraria montana var. lobata	0.10
Cortaderia jubata	0.01
Polygonum perfoliatum	0.01
Lygodium japonicum	0.01
Parthenium hysterophorus	0.01
Alternanthera philoxeroides	0.01

Grassland ecosystems

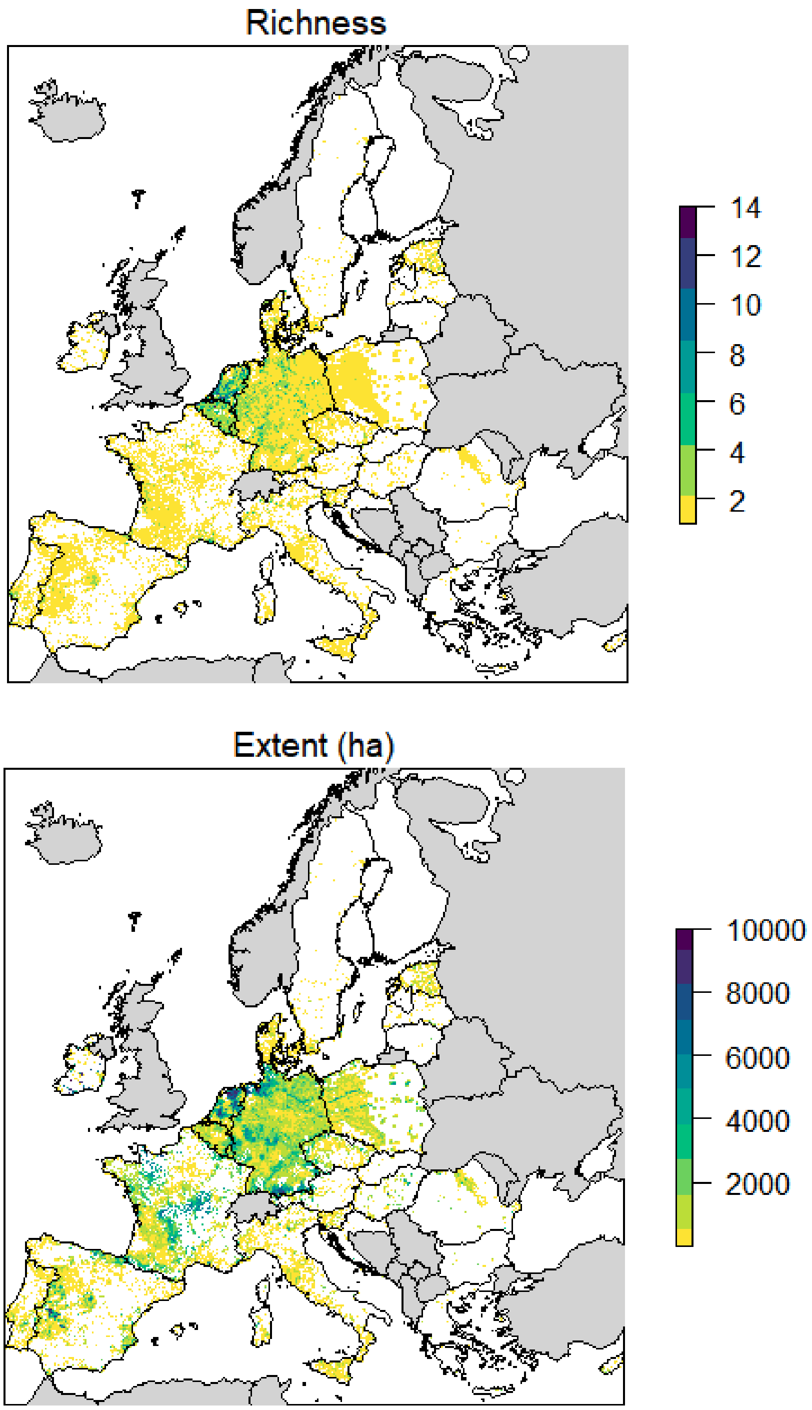

Figure S 4: Number of reported IAS (top) and extent of invaded areas (bottom) across grassland ecosystems. Extent refers to invaded hectares within the 100-km^2 grid cell. Countries beyond the geographic scope of the study are in light grey.

Table S 9: Relative extent (%) of invaded cropland ecosystems by animal IAS, in descending order.

Species	*Extent (%)*
Procyon lotor	28.53
Alopochen aegyptiacus	22.02
Myocastor coypus	14.25
Ondatra zibethicus	11.31
Vespa velutina nigrithorax	9.47
Procambarus clarkii	5.05
Muntiacus reevesi	0.66
Tamias sibiricus	0.52
Arthurdendyus triangulatus	0.38
Threskiornis aethiopicus	0.30
Nyctereutes procyonoides	0.22
Oxyura jamaicensis	0.21
Corvus splendens	0.19
Sciurus carolinensis	0.08
Callosciurus erythraeus	0.05
Herpestes javanicus	0.04
Acridotheres tristis	0.01
Nasua nasua	0.01
Sciurus niger	< 0.01

Table S 10: Relative extent (%) of invaded urban ecosystems by plant IAS, in descending order.

Species	*Extent (%)*
Ailanthus altissima	2.40
Impatiens glandulifera	1.11
Cabomba caroliniana	0.85
Asclepias syriaca	0.68
Heracleum mantegazzianum	0.43
Acacia saligna	0.39
Baccharis halimifolia	0.30
Heracleum sosnowskyi	0.22
Pennisetum setaceum	0.20
Gunnera tinctoria	0.05
Lysichiton americanus	0.04
Pueraria montana var. lobata	0.01
Prosopis juliflora	0.01
Heracleum persicum	0.01
Alternanthera philoxeroides	0.01
Polygonum perfoliatum	0.01
Andropogon virginicus	< 0.01
Cortaderia jubata	< 0.01
Lygodium japonicum	< 0.01
Parthenium hysterophorus	< 0.01
Gymnocoronis spilanthoides	< 0.01
Humulus scandens	< 0.01

Forest and woodland ecosystems

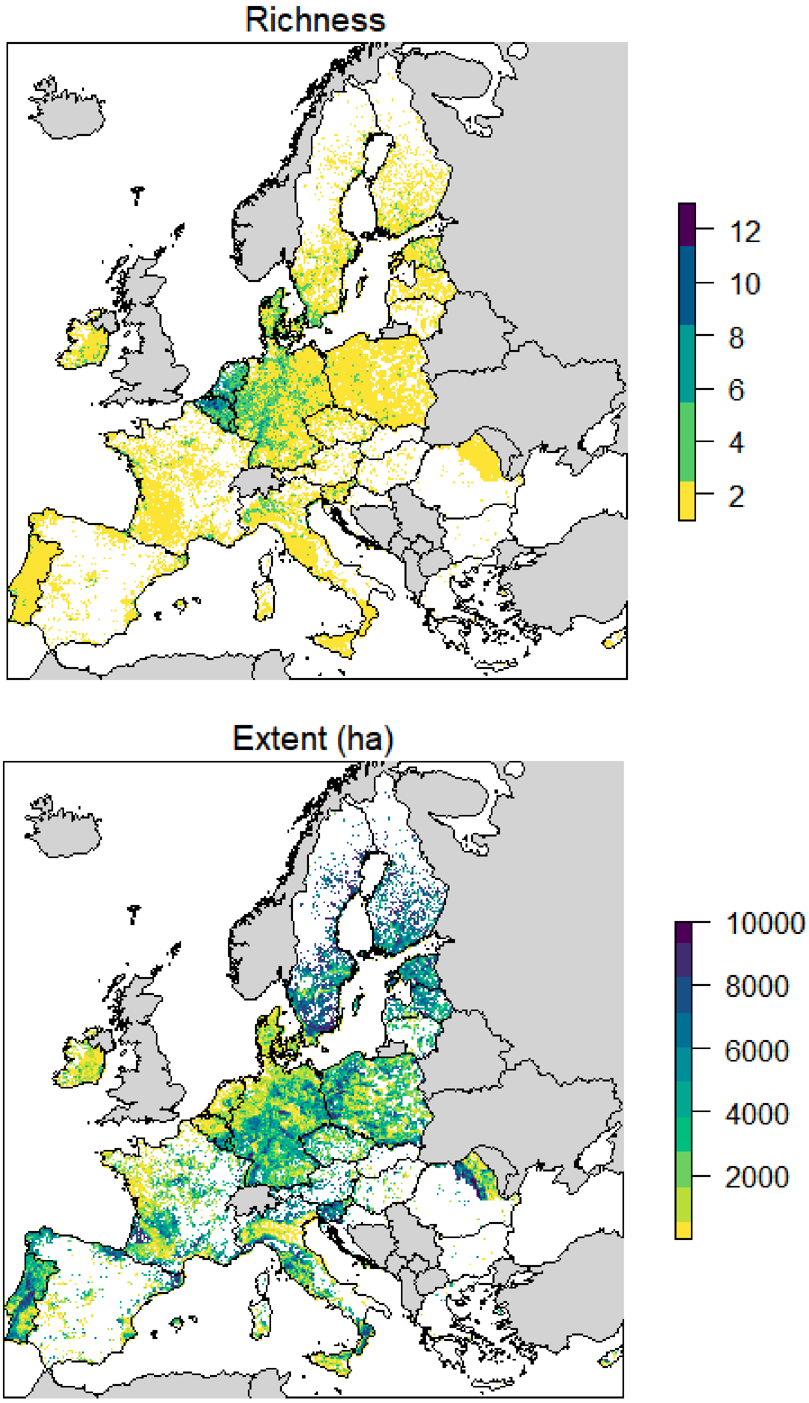

Figure S 5: Number of reported IAS (top) and extent of invaded areas (bottom) across forest and woodland ecosystems. Extent refers to invaded hectares within the 100-km^2 grid cell. Countries beyond the geographic scope of the study are in light grey.

Table S 11: Relative extent (%) of invaded forest and woodland ecosystems by animal IAS, in descending order.

Species	*Extent (%)*
Procyon lotor	14.76
Alopochen aegyptiacus	8.57
Myocastor coypus	8.03
Vespa velutina nigrithorax	5.79
Ondatra zibethicus	4.63
Nyctereutes procyonoides	4.62
Threskiornis aethiopicus	0.99
Oxyura jamaicensis	0.88
Sciurus carolinensis	0.55
Tamias sibiricus	0.16
Muntiacus reevesi	0.15
Corvus splendens	0.08
Herpestes javanicus	0.08
Callosciurus erythraeus	0.07
Acridotheres tristis	0.06
Nasua nasua	0.05
Sciurus niger	0.04
Arthurdendyus triangulatus	0.01

Table S 12: Relative extent (%) of invaded forest and woodland ecosystems by plant IAS, in descending order.

Species	*Extent (%)*
Impatiens glandulifera	20.30
Heracleum sosnowskyi	9.79
Heracleum mantegazzianum	8.29
Ailanthus altissima	7.84
Heracleum persicum	1.35
Lysichiton americanus	0.95
Baccharis halimifolia	0.72
Asclepias syriaca	0.55
Acacia saligna	0.32
Pueraria montana var. lobata	0.11
Gunnera tinctoria	0.09
Pennisetum setaceum	0.08
Humulus scandens	0.04
Andropogon virginicus	0.02
Cortaderia jubata	0.01
Alternanthera philoxeroides	< 0.01
Gymnocoronis spilanthoides	< 0.01
Polygonum perfoliatum	< 0.01
Lygodium japonicum	< 0.01
Prosopis juliflora	< 0.01
Parthenium hysterophorus	< 0.01

Heathland and shrub ecosystems

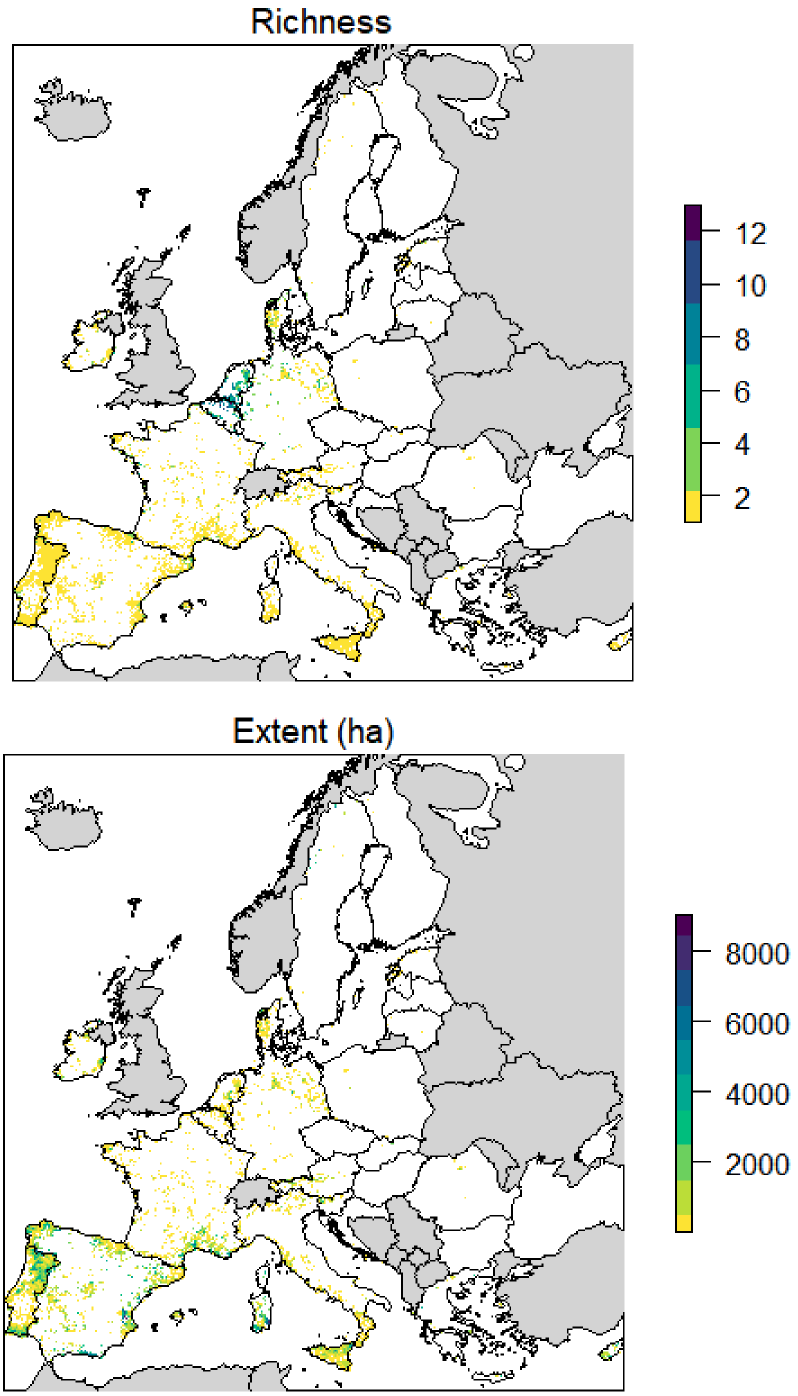

Figure S 6: Number of reported IAS (top) and extent of invaded areas (bottom) heathland and shrub ecosystems. Extent refers to invaded hectares within the 100-km^2 grid cell. Countries beyond the geographic scope of the study are in light grey.

Table S 13: Relative extent (%) of invaded heathland and shrub ecosystems by animal IAS, in descending order.

Species	*Extent (%)*
Vespa velutina nigrithorax	12.75
Alopochen aegyptiacus	11.34
Myocastor coypus	9.61
Procyon lotor	3.65
Threskiornis aethiopicus	2.37
Ondatra zibethicus	1.97
Oxyura jamaicensis	1.02
Sciurus carolinensis	0.91
Nyctereutes procyonoides	0.87
Acridotheres tristis	0.52
Herpestes javanicus	0.39
Muntiacus reevesi	0.28
Tamias sibiricus	0.27
Nasua nasua	0.24
Arthurdendyus triangulatus	0.11
Corvus splendens	0.06
Callosciurus erythraeus	0.05
Sciurus niger	< 0.01

Table S 14: Relative extent (%) of invaded heathland and shrub ecosystems by plant IAS, in descending order.

Species	*Extent (%)*
Ailanthus altissima	34.69
Acacia saligna	5.28
Impatiens glandulifera	4.27
Baccharis halimifolia	3.73
Heracleum mantegazzianum	2.76
Pennisetum setaceum	0.87
Gunnera tinctoria	0.68
Heracleum persicum	0.65
Heracleum sosnowskyi	0.30
Asclepias syriaca	0.15
Lysichiton americanus	0.11
Prosopis juliflora	0.04
Pueraria montana var. lobata	0.03
Cortaderia jubata	0.01
Alternanthera philoxeroides	0.01
Polygonum perfoliatum	< 0.01
Humulus scandens	< 0.01

Sparsely vegetated land ecosystems

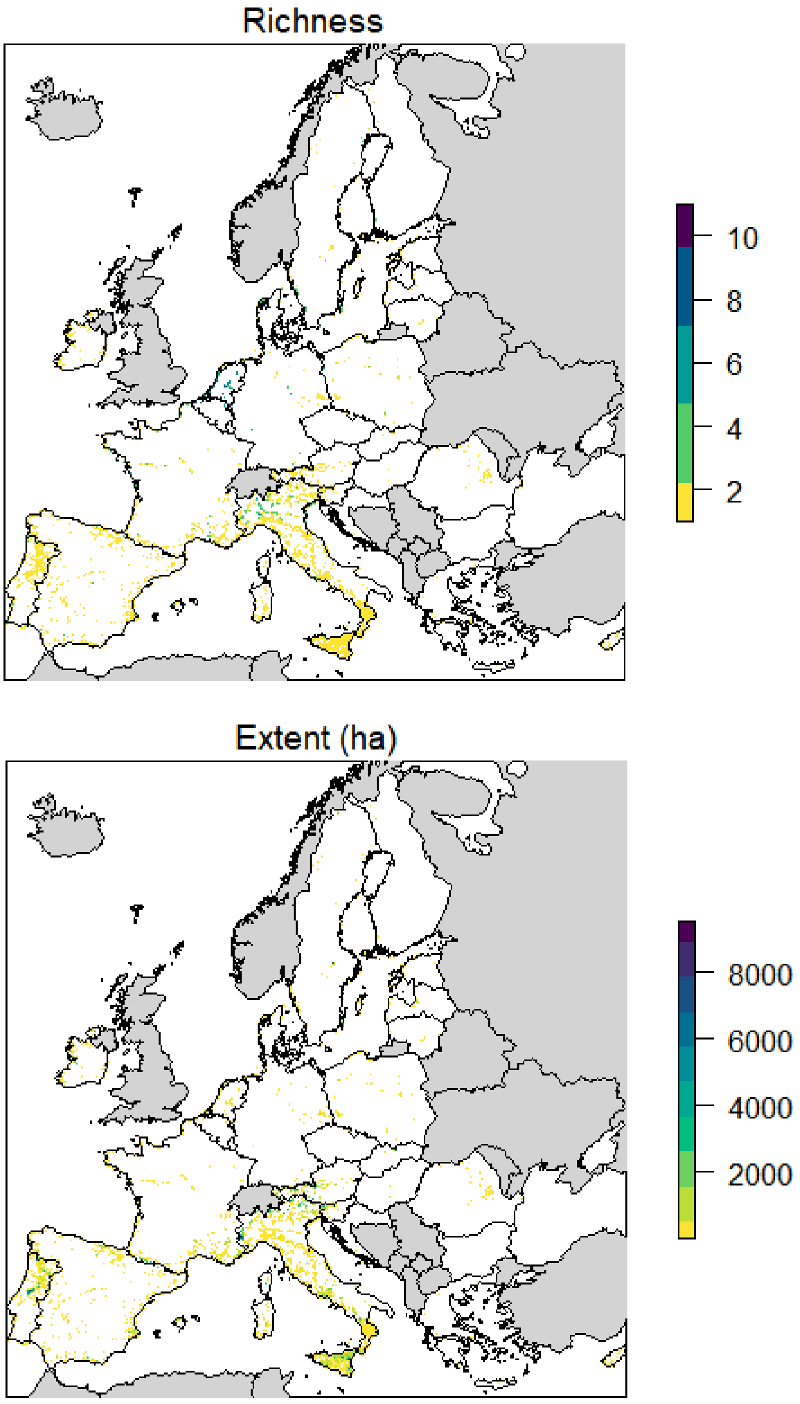

Figure S 7: Number of reported IAS (top) and extent of invaded areas (bottom) across sparsely vegetated land ecosystems. Extent refers to invaded hectares within the 100-km^2 grid cell. Countries beyond the geographic scope of the study are in light grey.

Table S 15: Relative extent (%) of invaded sparsely vegetated land ecosystems by animal IAS, in descending order.

Species	*Extent (%)*
Alopochen aegyptiacus	17.21
Myocastor coypus	14.07
Vespa velutina nigrithorax	5.66
Procyon lotor	3.06
Threskiornis aethiopicus	2.87
Oxyura jamaicensis	2.47
Ondatra zibethicus	1.72
Sciurus carolinensis	1.40
Nyctereutes procyonoides	0.87
Tamias sibiricus	0.64
Nasua nasua	0.38
Herpestes javanicus	0.27
Arthurdendyus triangulatus	0.23
Acridotheres tristis	0.22
Corvus splendens	0.14
Muntiacus reevesi	0.07
Callosciurus erythraeus	0.01

Table S 16: Relative extent (%) of invaded sparsely vegetated land ecosystems by animal IAS, in descending order.

Species	*Extent (%)*
Impatiens glandulifera	17.30
Ailanthus altissima	16.36
Heracleum mantegazzianum	4.99
Baccharis halimifolia	3.20
Acacia saligna	1.95
Heracleum sosnowskyi	1.36
Gunnera tinctoria	1.32
Pennisetum setaceum	0.70
Asclepias syriaca	0.56
Lysichiton americanus	0.45
Heracleum persicum	0.24
Pueraria montana var. lobata	0.14
Humulus scandens	0.07
Prosopis juliflora	0.03
Cortaderia jubata	0.01
Gymnocoronis spilanthoides	< 0.01

Freshwater ecosystems

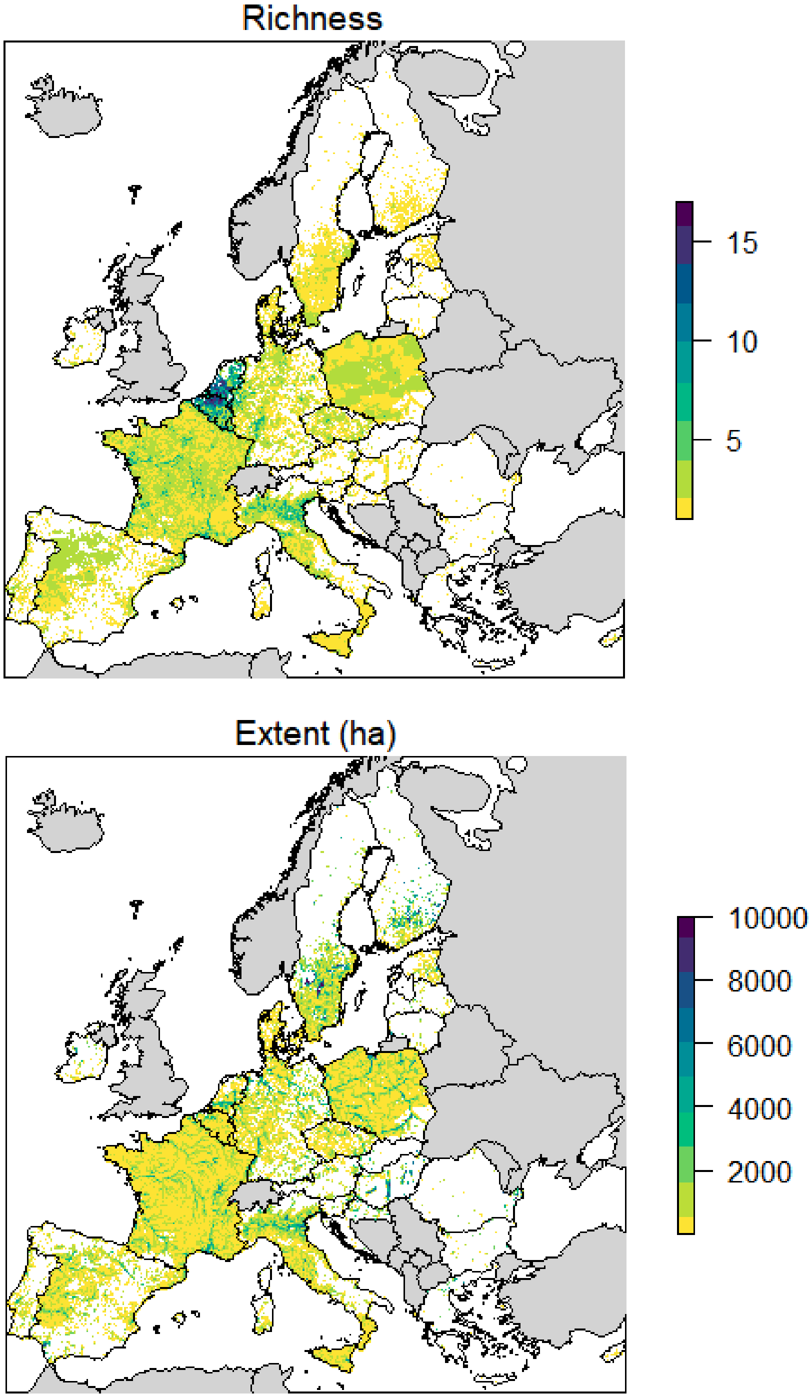

Figure S 8: Number of reported IAS (top) and extent of invaded areas (bottom) across freshwater ecosystems. Extent refers to invaded hectares within the 100-km^2 grid cell. Countries beyond the geographic scope of the study are in light grey.

Table S 17: Relative extent (%) of invaded freshwater ecosystems by animal IAS, in descending order.

Species	*Extent (%)*
Trachemys scripta	12.72
Orconectes limosus	12.69
Myocastor coypus	10.03
Pacifastacus leniusculus	9.59
Alopochen aegyptiacus	9.20
Pseudorasbora parva	8.03
Procambarus clarkii	7.49
Ondatra zibethicus	4.73
Threskiornis aethiopicus	3.25
Lepomis gibbosus	3.20
Eriocheir sinensis	2.96
Oxyura jamaicensis	2.35
Lithobates catesbeianus	0.84
Perccottus glenii	0.76
Orconectes virilis	0.10
Procambarus fallax f. virginalis	0.10

Table S 18: Relative extent (%) of invaded freshwater ecosystems by plant IAS, in descending order.

Species	*Extent (%)*
Elodea nuttallii	2.74
Ludwigia grandiflora	2.59
Myriophyllum aquaticum	1.77
Ludwigia peploides	1.67
Hydrocotyle ranunculoides	1.10
Lagarosiphon major	0.76
Eichhornia crassipes	0.70
Cabomba caroliniana	0.40
Myriophyllum heterophyllum	0.18
Salvinia molesta	0.04
Gymnocoronis spilanthoides	0.01
Alternanthera philoxeroides	0.01

Cited bibliography

1. EU. Commission Implementing Regulation (EU) 2019/1262 of 25 July 2019 amending Implementing Regulation (EU) 2016/1141 to update the list of invasive alien species of Union concern. *Off. J. Eur. Union* **L 199,** 1–4 (2019).
